# Single-cell analysis identifies PLK1 as a driver of immunosuppressive tumor microenvironment in LUAD

**DOI:** 10.1371/journal.pgen.1011309

**Published:** 2024-06-17

**Authors:** Yifan Kong, Chaohao Li, Jinpeng Liu, Sai Wu, Min Zhang, Derek B. Allison, Faisal Hassan, Daheng He, Xinyi Wang, Fengyi Mao, Qiongsi Zhang, Yanquan Zhang, Zhiguo Li, Chi Wang, Xiaoqi Liu

**Affiliations:** 1 Department of Toxicology and Cancer Biology, University of Kentucky, Lexington, Kentucky, United States of America; 2 Markey Cancer Center, University of Kentucky, Lexington, Kentucky, United States of America; 3 Department of Pathology and Laboratory Medicine, University of Kentucky, Lexington, Kentucky, United States of America; 4 Department of Biostatistics, University of Kentucky, Lexington, Kentucky, United States of America; Brigham and Women’s Hospital Department of Medicine, UNITED STATES

## Abstract

PLK1 (Polo-like kinase 1) plays a critical role in the progression of lung adenocarcinoma (LUAD). Recent studies have unveiled that targeting PLK1 improves the efficacy of immunotherapy, highlighting its important role in the regulation of tumor immunity. Nevertheless, our understanding of the intricate interplay between PLK1 and the tumor microenvironment (TME) remains incomplete. Here, using genetically engineered mouse model and single-cell RNA-seq analysis, we report that PLK1 promotes an immunosuppressive TME in LUAD, characterized with enhanced M2 polarization of tumor associated macrophages (TAM) and dampened antigen presentation process. Mechanistically, elevated PLK1 coincides with increased secretion of CXCL2 cytokine, which promotes M2 polarization of TAM and diminishes expression of class II major histocompatibility complex (MHC-II) in professional antigen-presenting cells. Furthermore, PLK1 negatively regulates MHC-II expression in cancer cells, which has been shown to be associated with compromised tumor immunity and unfavorable patient outcomes. Taken together, our results reveal PLK1 as a novel modulator of TME in LUAD and provide possible therapeutic interventions.

## Author summary

Our research has pinpointed a vital protein, PLK1, in the development of lung cancer. Through the use of mouse models simulating the onset of lung cancer and genetic sequencing analysis, we’ve discovered that high level of PLK1 hinders the immune response, making it challenging for the patient’s immune system to effectively eliminate lung cancer. Elevated PLK1 level specifically increases the presence of M2 macrophages, a type of immune cell, which, in turn, prevents the immune system from recognizing and attacking lung cancer cells. We’ve identified that this immune-suppressing effect is linked to PLK1’s role in secreting a specific molecule called CXCL2. This molecule promotes the presence of M2 macrophages and hampers the recognition of cancer cells by decreasing the machinery that presents the cancer cells’ signature to the immune system, known as MHC-II. These findings suggest that drugs targeting PLK1 could be a promising strategy for treating lung cancer patients.

## Introduction

Lung cancer accounts for one-fifth of all cancer mortalities in the US, making it the leading cause of cancer-related deaths [1]. Lung adenocarcinoma (LUAD) is the major type of this disease, comprising almost half of all cases [2]. LUAD is characterized by a myriad of genetic alternations that drive tumor heterogeneity, such as the common mutation forms involved in receptor tyrosine kinase pathways including EGFR, KRAS, ALK, and ROS1 genes [3]. These mutations exert a profound influence on disease progression and wield considerable sway over patients’ responses to treatment. As a result, a slew of targeted therapies has emerged, tailored to the unique genetic profiles of individual patients. Despite the advances in treatment options, the outlook for LUAD remains dishearteningly bleak, with the 5-year survival rate for metastatic disease is less than 5%. Therefore, precise early screening and the development of more efficacious treatment modalities are essential to improve patients’ outcomes.

Recently, immunotherapies have emerged as a novel treatment option for LUAD [4]. While this marks the new era of cancer treatment, the success of immunotherapies depends on several factors, including the patient’s immune status, and most importantly, the characteristics of the tumor microenvironment (TME), which has emerged as a pivotal area of cancer research [5]. TME is composed of various types of tumor-infiltrating myeloid cells and lymphocytes, and the interactions between cancer cells and these immune cells shape the properties of multiple cancer hallmarks, such as tumor growth, invasion, and metastasis. More closely, immunosuppressive TME can render immunotherapies ineffective, and this nature accounts for the fact that most cancer patients don’t respond to immunotherapy [6]. Within the realm of lung cancer, only 20% of patients respond to monotherapy with checkpoint blockade, a figure that modestly escalates to 40% when paired with conventional chemotherapy [7,8], underscoring a more complex yet underexplored features of TME in LUAD. Therefore, a better understanding of the TME holds paramount importance in developing effective immunotherapies.

Polo-like kinase 1 (PLK1) emerges as a pivotal serine/threonine kinase, orchestrating a multitude of crucial cellular processes. Its canonical function takes center stage in the intricate regulation of cell division across multiple phases [9–14]. Beyond its canonical role, studies have unveiled its participation in non-mitotic functions, including the regulation of DNA damage repair, metabolism, and immune response [15]. Dysregulation of PLK1, characterized by heightened expression levels, has been identified as a pivotal player in the initiation and progression of various cancer types [16]. Considering its importance in tumorigenesis, novel therapies targeting PLK1 have been developed with observable efficacy in cell and animal models [17]. Both our lab and others have proven that PLK1 overexpression in LUAD leads to cancer progression and is associated with poor overall survival [18,19]. Interestingly, recent studies have validated PLK1 as a potential target to improve the efficacy of immunotherapies in lung cancer [20], consistent with our findings in pancreatic cancer that targeting PLK1 enhances the effectiveness of immune checkpoint blockade [21]. While these results suggest that PLK1 may be an important modulator of tumor immunity, whether and how PLK1 functions in the TME of LUAD is largely unknown. Here, using single-cell RNA sequencing (scRNA-seq), we show that elevation of PLK1 incurs a suppressive TME in LUAD, marked by M2 polarization of tumor associated macrophages (TAM) and suppression of antigen presentation process. In addition, elevation of PLK1 attenuates the expression of class II major histocompatibility complex (MHC-II) in professional antigen presentation cells and cancer cells, leading to impaired antigen presentation and anti-tumor immunity. Overall, our results reveal PLK1 as a key regulator of TME and provide evidence of targeting PLK1 to improve immunotherapies in LUAD.

## Materials and methods

### Ethics statement

All animal experiments used in this study were approved by the University of Kentucky Division of Laboratory Animal Resources.

### Sample preparation for scRNA-seq

To perform scRNA-seq, 8-week-old LSL-*Kras*^G12D^/*Trp53*^fl/fl^ (KP) and LSL-*Kras*^G12D^/*Trp53*^fl/fl^/*Rosa26*^LSL-*Plk1*^ (KPP) mice (n = 3 each group), which were described previously [19], were intratracheally instilled with adenovirus-expressing Cre recombinase (Ad-Cre) at a viral titer of 2.5 × 10^7^ PFU per mouse according to the established protocol [22]. Tumor formations were monitored every week by MRI and mice were sacrificed after 12 weeks of Ad-Cre delivery. Single-cell suspension was prepared following 10x Genomics Cell Preparation Guide. Briefly, mice lung tissues were harvested and cut into smaller pieces, then we used pipette to extensively pipette tissues up and down to get cell mixture in DMEM medium containing 2% FBS. This mixture was filtered with a 70 μm nylon mesh strainer, centrifuged at 300 g for 10 mins, then resuspended in DMEM medium containing 2% FBS to get single-cell suspension. The single cell suspension was further sorted by cell size to enrich immune cell populations, and this suspension was processed for scRNA-seq. Cell viability was at least 95% for all samples. 10000 cells were targeted for sequencing and loaded onto a Chromium Controller (10x Genomics) for gel beads-in-emulsion formation. Library preparation was conducted using Chromium Next GEM Single Cell 3’ Gene Expression Kit (v3.1, 10x Genomics) according to the manufacturer’s instructions. Single indexed, paired-end libraries were sequenced on an HiSeq 2500 sequencer (Illumina).

### Analysis of scRNA-seq

scRNA-seq gene expression libraries were mapped to mm10 mouse reference (10x Genomics pre-built reference, mm10-2020-A) using Cell Ranger (v6.1.1, 10x Genomics). For each library, Cell Ranger filtered matrix was subjected to QC filters to remove low quality cells with ≤ 500 features or percentage of mitochondrial transcripts ≥ 15. DoubletFinder analysis was then performed on each library separately to identify and filter potential doublets with default parameters [23]. Additionally, cells with features > 8000 or counts > 50,000 were removed. Pass QC cells from the KP and KPP groups were combined and processed using the Seurat (v3) R package [24]. Count matrices were Log-normalized and scaled. The top 2,000 highly variable genes were selected to define principal components (PCs). Batch integration was performed via Harmony algorithm (v0.1.0) to the combined data for batch effect corrections with default settings [25]. Neighbor analysis was performed by FindNeighbors function using the PCs from the Harmony dimension. Immune cell clusters were identified with FindClusters function. UMAP was calculated based on the Harmony dimension for clusters visualization. Identification of cluster markers was performed using a Wilcoxon rank-sum test by comparing each cluster with the rest of the cells. For differential gene expression analysis, we used FindAllMarkers function in Seurat to identify the up/down-regulated sets of genes between different groups in the cell populations. For calculating M1 and M2 module scores, we used AddModuleScore function in Seurat to add the M1 and M2 module scores for each individual cell. Gene Set Enrichment Analysis (GSEA) was performed with the differentially expressed gene list generated by FindAllMarkers function and fgsea R package [26], and we used the human KEGG and HALLMARK datasets [27,28].

### Analysis of public dataset and bulk RNA-seq

For STRING analysis [29], genes functionally associated with CXCL2 were directly checked with online STRING tool (https://string-db.org/). For gene expression, correlation, and survival analyses of TCGA-LUAD dataset, patients’ demographic data with RSEM and zscore values (normalized to all samples) of RSEM were downloaded from cBioportal [30–32]. For gene expression analysis of PLK1 and MHC-II genes, zscore values (normalized to all samples) of RSEM were used. For correlation analysis between PLK1 and MHC-II genes, Log_2_RSEM were used for plotting. For survival analysis, the MHC-II signature scores were calculated by averaging the Log_2_RSEM+1 values of human MHC-II genes, and the median was used to separate patients into MHC-II High (> median) and MHC-II Low (< = median) groups. For immune deconvolution of TCGA-LUAD bulk RNA-seq, patients with TP53 mutant and KRAS^G12D/S/C/A/V^ or KRAS^G13C^ mutant were selected and their gene expression matrix was used as the input in CIBERSORT algorithm [33], using online platform and the default LM22.txt immune cell gene signature (https://cibersortx.stanford.edu/). For each patient, the M1/M2 ratio was calculated. Analysis of bulk RNA-seq data from KP and KPP tumors was described previously [19].

### Cell culture

KP and KPP cell lines were isolated from transgenic mice instilled with Ad-Cre at 8-week-old and 12–14 weeks after Ad-Cre infection, which was described before [19]. H358 cell line was purchased from ATCC (CRL-5807). All cell lines were cultured in RPMI 1640 medium containing 10% FBS, 1% penicillin-streptomycin at 37°C incubator with 5% CO_2_. For preparation of conditioned medium, KP or KPP cells were cultured in 100 mm dishes until 70–80% confluency, then medium was refreshed with DMEM medium containing 0.5% FBS and continued to culture for 48 hours. The resulting conditioned medium was used for coculture experiments and mouse cytokine array. For transfection of siRNA, predesigned siRNA (Sigma) targeting PLK1 was transfected with jetPRIME Versatile DNA/siRNA transfection reagent (Polyplus, 101000001) according to the manufacturer’s instructions. 48 hours after transfection, cells were harvested for subsequent experiments. All the cell lines were within 50 passages and tested negative for mycoplasma contamination.

### Coculture experiments

Macrophages and dendritic cells were isolated from the bone marrows of C57/BL6 mice (The Jackson Laboratory, 000664). Briefly, bone marrows were flushed out of the femur and tibia with complete DMEM medium using 25-gauge needles. After centrifugation at 400 g for 5 mins, bone marrows suspension was treated with 1x RBC lysis buffer (Thermo, 00-4333-57) and centrifuged again. The cell pellets were then washed with 1x PBS twice and resuspended in refresh complete DMEM medium. Cells were cultured at 37°C incubator with 5% CO_2_ for a week to induce differentiation. For induction of macrophages, cells were treated with 10 ng/ml mouse *Csf1*. For induction of dendritic cells were treated with 10 ng/ml mouse *Csf2* and 20 ng/ml mouse *Il-4*. Medium with cytokines was refreshed every two days. For coculture with cells, KP or KPP cells were directly seeded onto the same plates with immune cells at indicated ratios for 48 hours. Treatment started at 6 hours post-coculture and continued until the end of experiments. For coculture with conditioned medium, the conditioned medium from KP or KPP was used to replace normal culture medium for immune cells and continued to culture for 48 hours.

### Flow cytometry

Cells were harvested by a cell scraper and washed once with 1x PBS, then stained on ice with indicated antibodies in 1x PBS for 30 mins. Samples containing tumor cells were fixed with 70% ethanol before staining. Data were acquired on a BD Symphony A3 analyzer (BD Biosciences) and analyzed using FlowJo software (V10.10.0, BD Biosciences). Gating strategy can be found in **S1 Appendix**.

### Mouse cytokines array

Mouse cytokine array experiment was performed using Proteome Profiler Mouse Cytokine Array Kit (R&D Systems, ARY006) according to the manufacturer’s instructions. Briefly, conditioned medium from KP and KPP was incubated with membranes containing pre-adsorbed primary antibodies targeting mouse cytokines. Following wash step, the membranes were then incubated with HRP-linked secondary antibodies. After washing membranes again, membranes were probed with chemiluminescent reagents to visualize cytokine spots. Medium containing 0.5% FBS but without cancer cells was used as the negative control. Images were captured with ChemiDoc Imaging System (Bio-Rad) and analyzed with Image Lab software (Bio-Rad).

### Immunohistochemistry (IHC)

IHC staining was performed with formalin-fixed, paraffin-embedded slides prepared from KP and KPP tumors and the VECTASTAIN Elite ABC Universal PLUS Kit (Vector Laboratories, PK-8200) according to the manufacturer’s instructions. Staining was visualized with ImmPACT DAB Substrate Kit (Vector Laboratories, SK-4105) and counterstained with Harris’s hematoxylin. Images were taken with a Nikon microscope and analyzed by Fiji software [34].

### Statistical analysis

Statistical analyses were performed with the statistical functions in GraphPad Prism 8 and R programming language. Unless denoted elsewhere, an unpaired two-sided t test was used as the default method for numeric results, excepting the survival analysis which was performed with a Log-rank test. Normality and variance of results were checked to confirm the compliance of t test. Statistical significance was set at p < 0.05 unless denoted elsewhere.

Antibodies, chemicals, and siRNA information can be found in **S8 Table**.

## Results

### Elevation of PLK1 is associated with immunosuppressive TME in LUAD

**Fig 1:**
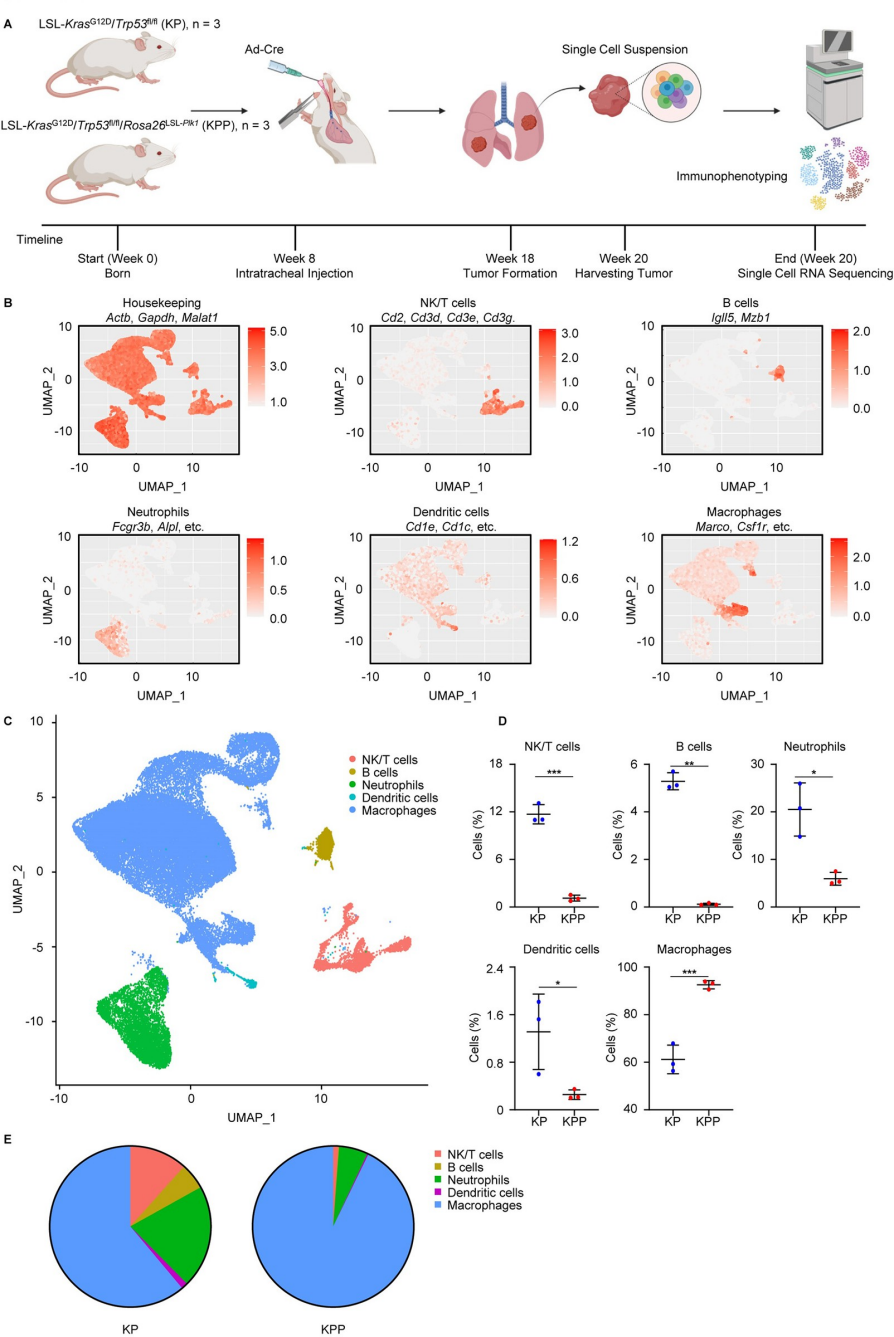
scRNA-seq analysis of KP and KPP mice. **A,** Workflow of scRNA-seq of KP and KPP mice. Created with BioRender.com. **B,** Feature plots of marker genes used for classification of NK/T cells, B cells, neutrophils, dendritic cells, macrophages. The full gene list for neutrophils is *Fcgr3b*, *Alpl*, *Cxcr1*, *Cxcr2*, *Adgrg3*, *Cmtm2*, *Prok2*, *Mme*, *Mmp25*. The full gene list for dendritic cells is *Cd1e*, *Cd1c*, *Fcer1a*, *Pklb*, *Cyp2s1*, *Ndrg2*. The full gene list for macrophages is *Marco*, *Csf1r*, *Cd68*, *Gldn*, *Apoe*, *Cdl3l1*, *Trem2*, *C1qb*, *Nupr1*, *Folr*. Color scale represents the average expression of marker genes. **C,** UMAP of major immune cell populations in KP and KPP. **D,** Comparison of cell populations between KP and KPP (n = 3). Data are shown as mean ± SD. **E,** Proportion of cell populations in KP and KPP. *, p < 0.05. **, p < 0.01. ***, p < 0.001.

To investigate the impact of PLK1 on lung cancer TME, we designed a workflow to perform the scRNA-seq using our KP and KPP mouse models (**Fig 1A**), which we previously reported [19]. Using the markers to differentiate non-immune and immune cells, the results showed that most cells (over 95%) were captured as immune cells without significant abundance differences between KP and KPP groups (**S1A–S1C Fig**), demonstrating the efficacy of scRNA-seq to enrich immune cells and the reliability to characterize TME. We started by unbiased clustering of all immune cells and identified 20 different populations characterized by a specific gene signature (**S1D and S1E Fig and S1 Table**), indicating a significant intratumoral heterogeneity of immune cell populations. Notably, the different proportions of immune cell clusters suggested a distinct TME in KP and KPP (**S1F and S1G Fig**). To better visualize the differences in immune cell populations, we refined our clustering strategy and performed differentially expressed gene analysis of all clusters and used well-established gene signatures of major immune cells to assign the cell type of each cluster (**Fig 1B and 1C and S2 Table**). After cell type identification, we found that KPP tumors had significantly lower proportions of all immune cell types except for TAM compared to KP tumors (**Fig 1D and 1E**). In both groups, TAM were the major population, suggesting the unique function of them in lung cancer progression. Besides, the lower levels of tumor-infiltrating lymphocytes (B cells and NK/T cells) in KPP tumors indicated a more immunosuppressive TME in this group. Since T cells and NK cells are the major immune cells exerting direct anti-tumor immunity, we further clustered the NK/T subpopulation in KP and KPP tumors using the empirical marker genes (**S2A and S2B Fig and S3 Table**). Clearly, KPP tumors displayed decreased proportions of tumor-infiltrating NK cells and T cells of all subtypes (**S2C Fig**). The lower levels of them in KPP tumors were impactful and further supported a colder TME in this group, as NK/T cells were the major immune cell compartments responsible for eliminating tumors. This observation suggested that KPP tumors were specifically protected from the anti-tumor immunity and thus resistant to the host immune defense. Taken together, these results illustrated that high PLK1 in LUAD promoted a suppressive immunophenotype.

### Tumor-promoting M2-like macrophages propagate in high-PLK1 TME of LUAD

**Fig 2:**
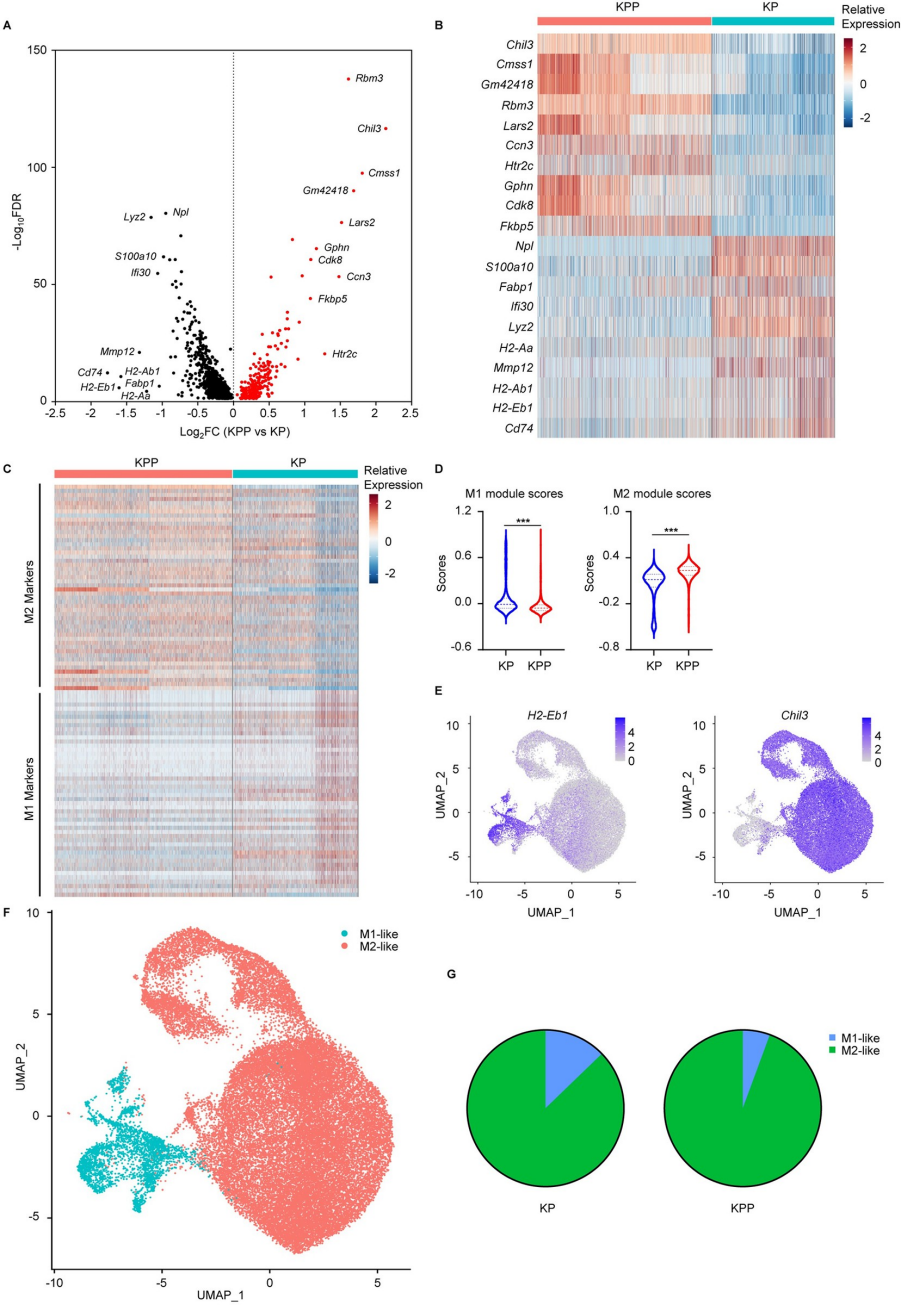
High PLK1 is associated with increased M2 macrophages. **A,** Volcano plot of differentially expressed genes (KPP vs KP, FDR < 0.05) in TAM. Top 10 up- and down-regulated genes (ranked by Log_2_FC) are labeled. Log_2_FC, Log_2_fold change. Also see S4 Table for full gene list. **B,** Heatmap of top 10 up and down-regulated genes identified in A. **C,** Heatmap of top 50 representative genes (ranked by Log_2_FC) in M1 and M2 gene signatures. Also see S5 Table for full gene list. **D,** M1 and M2 module scores of TAM calculated by AddModuleScore function of Seurat package, using the full M1 and M2 gene lists in S5 Table. Data are shown as mean ± SD. **E,** Feature plots of representative marker genes in C used for classification of M1-like (*H2-Eb1*) and M2-like (*Chil3*) macrophages. Color scale represents the relative expression levels of genes. **F,** UMAP of M1-like and M2-like macrophages in KP and KPP. **G,** Proportion of M1-like and M2-like macrophages in KP and KPP. ***, p < 0.001.

The relatively higher proportion of TAM in KPP tumors was intriguing, and this phenomenon enticed us to investigate the role of TAM in high-PLK1 TME. Given that the functions of TAM were dichotomous, we specially focused on the proportions of M1 and M2 macrophages in KP and KPP tumors, which are the most widely used macrophage subtypes and exert either tumor-suppressing or tumor-promoting functions, respectively. To identify the two functionally distinct macrophage subtypes, we first performed differentially expressed gene analysis in TAM and identified 1201 statistical significant genes between KP and KPP tumors (**Fig 2A and S4 Table**). Investigation of top 10 up- and down-regulated genes in KPP group revealed that TAM in KPP tumors were positively enriched for genes associated with M2 macrophages (e.g., *Chil3*, *Ccn3*) while negatively enriched for genes associated with M1 macrophages (e.g., *H2-Eb1*, *H2-Ab1*), suggesting that TAM in KPP tumors were more enriched for M2 subtype (**Fig 2B**). Since macrophages may express both M1 and M2 genes and two subtypes are somehow convertible, we used M1 and M2 gene signatures and calculated the module scores to better evaluate the functional state of macrophages in two groups. We found that macrophages in KPP expressed higher levels of M2-related genes and lower levels of M1-related genes as shown by expression heatmap and module scores (**Fig 2C and 2D and S5 Table**), further supporting increased M2 macrophages in KPP tumors. Since the representative genes (e.g., *H2-Eb1* and *Chil3*) in the M1 and M2 gene signature were able to separate the macrophage clusters (totally 11) clearly (**Fig 2E**), we then used the two gene signatures and assigned each macrophage cluster to either M1-like (higher M1 module scores) and M2-like (higher M2 genes) subtypes and the results indeed consolidated that TAM in KPP tumors consisted of more M2-like macrophages compared to KP tumors (**Fig 2F and 2G**). Given that M2 macrophages were considered as tumor-promoting and immunosuppressive, the higher proportion of M2-like macrophages in KPP tumors as indicated by scRNA-seq data was consistent with a colder TME in this group. In sum, these results supported the notion that high PLK1 was associated with increased tumor-promoting M2-like macrophages in LUAD.

### High PLK1 suppresses antigen presentation pathway and induces M2 polarization

**Fig 3:**
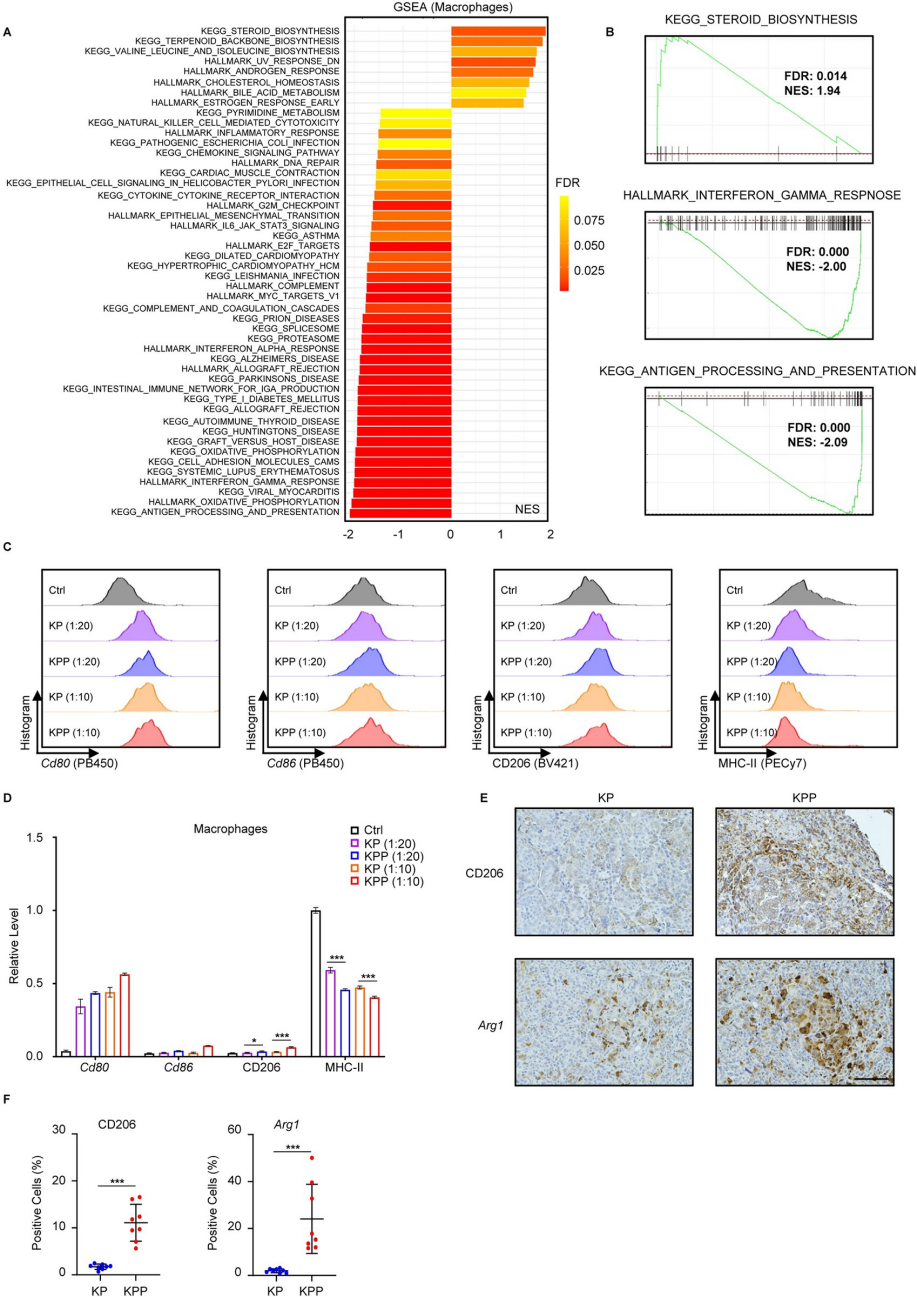
PLK1 suppresses antigen presentation pathway and promotes M2 polarization. **A,** Significant pathways (KPP vs KP, FDR < 0.1) altered in macrophages identified by GSEA. NES, normalized enrichment score. **B,** GSEA plots of three exemplary pathways in A. **C,** Flow cytometry analysis of indicated genes in BMDM cocultured with KP or KPP at the indicated ratio for 48 hours. **D,** Quantification of C (n = 3). The averages of *Cd80*/*Cd86*/CD206/MHC-II in the histogram of the gated populations are normalized to the average of MHC-II in Ctrl group and shown as Relative Level (mean ± SD). Also see S1 Appendix. **E,** Representative IHC images of KP and KPP tumors stained with M2 markers CD206 and *Arg1*. Scale bar, 100 μm. **F,** Quantification of E (n = 8). Data are shown as mean ± SD. *, p < 0.05. ***, p < 0.001.

Since high PLK1 was associated with increased infiltration of M2-like macrophages, we sought to delineate the biological consequence of this phenotype. To achieve this aim, we performed GSEA in TAM. The results showed that multiple pathways associated with the functions of macrophages were dramatically disturbed (**Fig 3A and Table A in S6 Table**). Several pathways related to immune response were suppressed in KPP tumors, such as pathways associated with inflammation (e.g., interferon gamma response, **Fig 3B**), pathogen defense (e.g., viral myocarditis), and autoimmune diseases (e.g., systemic lupus erythematosus). In addition, pathways associated with steroid and lipid metabolism were elevated in KPP tumors (e.g., steroid biosynthesis, **Fig 3B**), which were previously demonstrated to promote M2 polarization [35]. These results suggested an impaired anti-tumor immune response and were in congruent with the observations of more M2-like macrophages and a more immunosuppressive phenotype in KPP tumors. Of note, antigen processing and presentation pathway was among the most suppressed pathway in TAM (**Fig 3A and 3B and Table A in S6 Table**). Professional antigen presentation cells such as M1 macrophages rely on MHC-II and costimulatory factors for their antigen processing and presentation function. Indeed, review of gene expressions and macrophages markers (**Fig 2A–2C, and S4 and S5 Tables**) showcased that some low-expression M1 signature genes in TAM of KPP tumors were MHC-II genes (e.g., *Cd74*, *H2-Eb1*, *H2-Ab1*, *H2-Aa*) and costimulatory factors (e.g., *Cd86*, *Cd80*). Given that this pathway was key to successful anti-tumor immunity and is a feature of M1 macrophages [36], the lower expression of MHC-II and costimulatory factors, as well as loss of antigen presentation function, in TAM was consistent with the more M2-like macrophages in KPP group and the proposed immunosuppressive role of PLK1. Moreover, results of GSEA in other immune cells showed that dampened antigen processing and presentation was a shared consequence among immune cell populations (**S3 Fig and S6 Table**), suggesting that suppression of this critical pathway was a general consequence of high PLK1 expression. Based on these results, we hypothesized that PLK1 could inhibit antigen presentation pathway and promote M2 polarization. To validate our hypothesis, we first performed in vitro coculture experiments with established cell lines from KP and KPP mice and used flow cytometry to monitor expressions of important genes and markers in antigen presentation and macrophages polarization **(Fig 3C and 3D)**. Short-term coculture of bone marrow derived macrophages (BMDM) with KP or KPP cells showed that both cells elevated costimulatory factors (*Cd80* and *Cd86*) with much stronger effect from KPP cells. However, only KPP cells were able to induce higher level of M2 marker CD206, indicating the M2 polarization effect of PLK1. Coculture with both cell lines attenuated MHC-II expression, possibly due to the general cancer cells’ intrinsic immune suppression mechanism. Compared to KP, the level of MHC-II was lower in KPP group. Given that MHC-II genes play a pivotal role in antigen processing and presentation and they make up of M1 gene signature, the lower expression of MHC-II complex in KPP group confirmed that antigen presentation function was specifically dysregulated in high PLK1 condition, and this might be associated with increased M2 polarization. Furthermore, IHC staining confirmed that canonical M2 markers (CD206 and *Arg1*) were higher in the TME of KPP tumors (**Fig 3E and 3F**). Collectively, these data demonstrated that PLK1 suppressed antigen presentation pathway and induced M2 polarization in LUAD.

### Immunosuppressive function of PLK1 depends on CXCL2 and varies in different cell type

**Fig 4:**
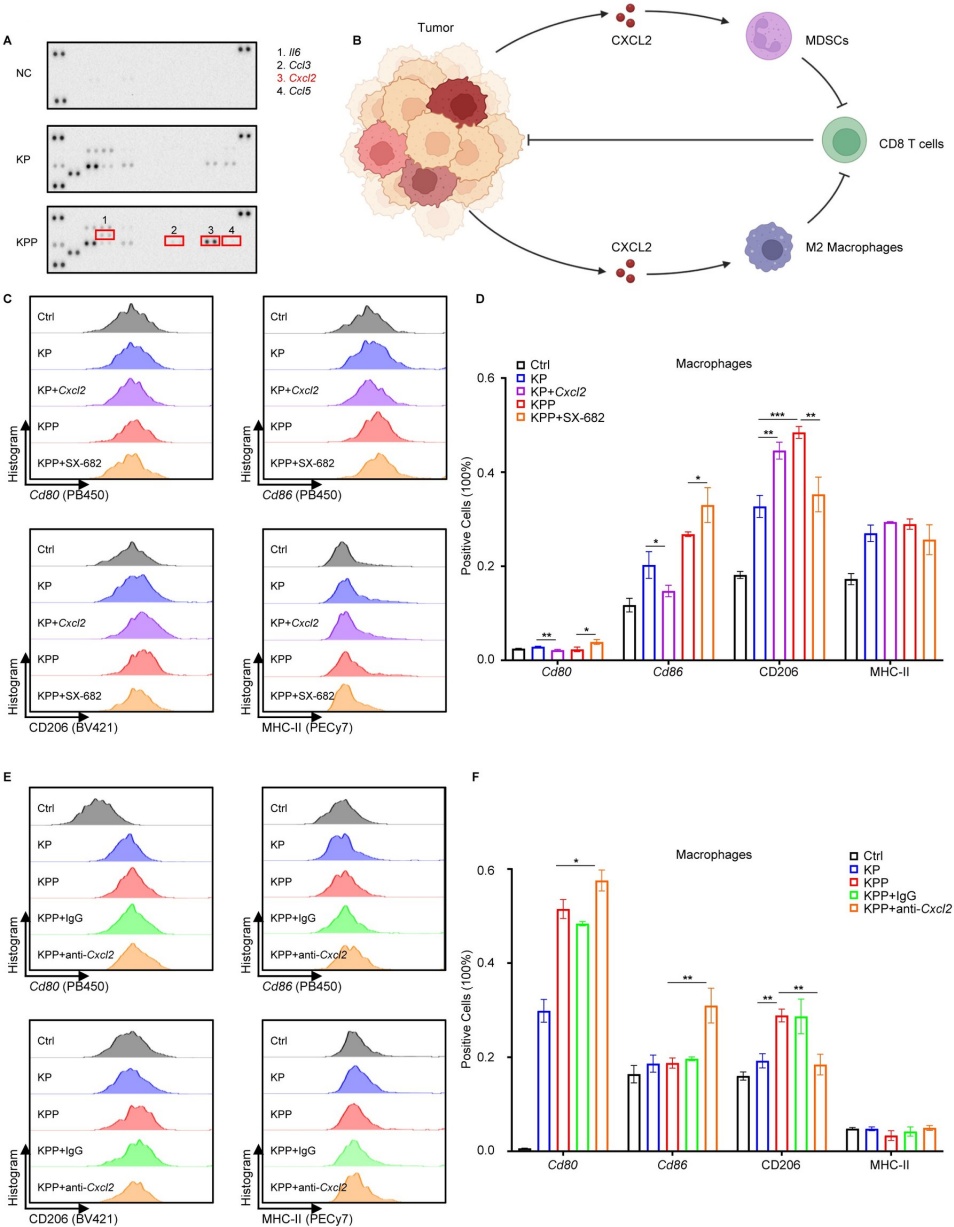
CXCL2 is responsible for the immunosuppressive function of PLK1. **A,** Cytokines array detection of cytokines secretion from the culture medium of KP and KPP. Notable differences are labeled. **B,** Illustration of CXCL2’s function in suppressing anti-tumor immunity. Created with BioRender.com. **C,** Flow cytometry analysis of BMDM cocultured with conditioned medium from KP or KPP for 48 hours, with or without recombinant *Cxcl2* (2 ug/ml) or CXCR1/CXCR2 inhibitor SX-682 (1 μM) treatment. **D,** Quantification of C (n = 3). The percentage of positive cells after gating is shown as mean ± SD. Also see S1 Appendix. **E,** Flow cytometry analysis of BMDM cocultured with conditioned medium from KP or KPP for 48 hours, with or without anti-*Cxcl2* neutralization antibodies (5 ug/ml) treatment. **F,** Quantification of E (n = 3). The percentage of positive cells after gating is shown mean ± SD. Also see S1 Appendix. *, p < 0.05. **, p < 0.01. ***, p < 0.001.

Paracrine signaling through the release of cytokines from cancer cells is critical for intercellular communication and shaping anti-tumor immunity [37]. Inspection of pathway analysis in TAM (**Fig 3A and Table A in S6 Table**) revealed that several cytokine and chemokine pathways were altered (e.g., interferon response pathway), and this led to our hypothesis that PLK1 exerted its immunosuppressive function via regulation of cytokine secretion. To test our hypothesis, we performed a cytokines array of mouse cytokines released to the conditioned medium of KP and KPP cells. We found that cytokine profiles were indeed distinct between KP and KPP. Compared to KP, KPP cells secreted more *Il6*, *Cxcl2* and *Ccl3* but less *Ccl5* (**Fig 4A**). The increased secretion of *Il6* was reasonable given its established role in M2 polarization [38]. Besides, *Ccl3* (macrophage inflammatory protein 1-alpha) was one of the cytokines released from macrophages and *Ccl5* was responsible for T cells chemotaxis [39,40], so their alternations were congruent with the higher proportion of macrophages and lower infiltration of T cells in KPP tumors. Among them, we were especially interested in *Cxcl2*, as it was the most highly secreted cytokine in the conditioned medium of KPP. It has been shown that secreted CXCL2 in TME can recruit myeloid derived suppressor cells and M2 macrophages [41,42], and this leads to suppression anti-tumor response (**Fig 4B**). We also investigated which pathway was responsible for the increased secretion of *Cxcl2* in KPP. A preliminary STRING pathway network analysis in human and mice identified several important genes related to CXCL2 (**S4A Fig**). Except for some known interaction genes (e.g., CXCR1 and CXCR2, two receptors for CXCL2), we found that in both species the genes related to JAK-STAT3 pathway (e.g., IL6/*Il6*) and NFKB pathway (e.g., TNF, *Rela*) commonly emerged. GSEA of RNA-seq data confirmed that two pathways were highly active in KPP cells (**S4B Fig**). Of note, although not very striking, *Il6* was simultaneously detected with *Cxcl2* in the secretion profile of KPP, which was not observed in KP control (**Fig 4A**). These results provided evidence that IL6/JAK/STAT3 and TNF/NFKB axis might account for the increased *Cxcl2* in KPP cells.

In mice, *Cxcl2* can bind to *Cxcr1* and *Cxcr2*, albeit with much higher affinity to *Cxcr2* [43]. Given that *Cxcl2* was predominant in KPP-secreted cytokines and its established role in recruiting M2 macrophages, we hypothesized that the observed immunosuppressive function of PLK1 depended on CXCL2. To test this assumption, we performed coculture experiments of BMDM with conditioned medium from KP or KPP (**Fig 4C and 4D**), treated with recombinant mouse *Cxcl2* or SX-682, a previously reported dual CXCR1/CXCR2 inhibitor in clinical trial [44]. Coculture of BMDM with conditioned medium recapitulated most of the results from coculturing with cells, but MHC-II was not decreased. Instead, MHC-II elevated under this condition, indicating that the decrease of MHC-II in coculture with cells directly came from interaction between cancer cells and immune cells. Coculture with conditioned medium still elevated *Cd80* and *Cd86* in both groups, with a much higher induction of M2 marker CD206 in KPP group. Intriguingly, the addition of *Cxcl2* in conditioned medium from KP was able to reduce *Cd80* and *Cd86* while increasing the level of CD206. Besides, adding CXCR1/CXCR2 inhibitor in conditioned medium from KPP group elevated *Cd80* and *Cd86* but attenuated CD206 expression. However, MHC-II was not disturbed by either *Cxcl2* or SX-682. Considering that mouse *Cxcl2* binds to *Cxcr2* with much higher affinity, and mouse *Cxcr1* mainly binds to other cytokines [43], the off-target effect of SX-682 in our experimental setting could not be ignored. To address this issue, we performed a parallel coculture experiment of BMDM with conditioned medium from KP and KPP (**Fig 4E and 4F**), treated with mouse IgG control or anti-*Cxcl2* neutralization antibodies. Treatment with neutralization antibodies witnessed an uptrend of *Cd80* and *Cd86* but a downtrend of CD206, with an unchanged status of MHC-II, reassuring the results obtained from the coculture experiment using SX-682. These data illustrated that *Cxcl2* was partially responsible for the effect of PLK1 in suppressing antigen presentation and inducing M2 polarization, mainly focused on regulation of costimulatory factors. As costimulatory factors in professional antigen presentation cells, the functions of CD80 and CD86 are similar in dendritic cells, and CD206 is also a hallmark of immature dendritic cells with incomplete immune functions [45]. Thus, detection of these markers in dendritic cells has similar value in dissecting the biological effect of PLK1 and CXCL2 on antigen presentation. We then repeated all three coculture experiments with dendritic cells isolated from bone marrow. The results revealed that alternations of *Cd80*, *Cd86* and CD206 were quite similar and repeatable, but the level of MHC-II displayed distinct changes. In the coculture experiment with KP/KPP cells and dendritic cells, an increased proportion of KP cells accompanied an elevated level of MHC-II, but an increased ratio of KPP cells reduced MHC-II expression (**S5A and S5B Fig**). This was different from the results in BMDM, where a higher ratio of both cell lines was associated with a lower level of MHC-II. However, in both settings, KPP cells were capable of diminishing MHC-II expression more strongly compared to KP, suggesting PLK1’s universal suppression of antigen presentation by limiting MHC-II expression. In the coculture experiment with KP/KPP conditioned medium and dendritic cells, the results demonstrated that the effect of PLK1 on MHC-II could be explained by CXCL2, as treatment with *Cxcl2* in KP group reduced MHC-II expression and treatment with either SX-682 or neutralization antibodies in KPP group elevated MHC-II level (**S5C, S5D, S5E and S5F Fig**), which was different from the results in macrophages. Despite these differences, the disturbance of antigen presentation and induction of immunosuppressive environment by CXCL2 was observed. Taken together, these results stated that CXCL2 accounted for the immunosuppressive function of PLK1 in LUAD, but the actual effect of CXCL2 might vary in different immune cells.

### PLK1 negatively regulates MHC-II in cancer cells

**Fig 5:**
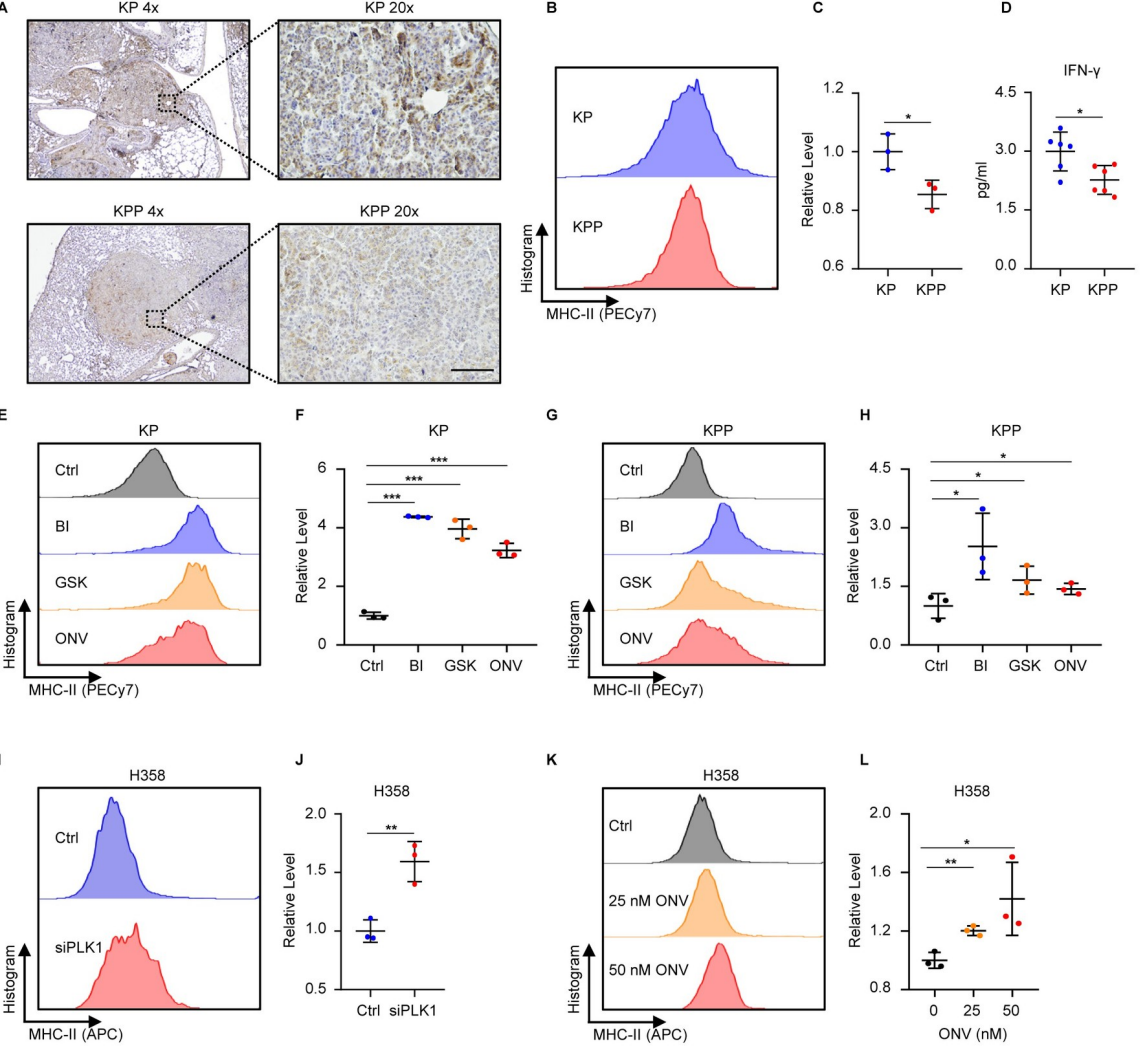
PLK1 suppresses MHC-II expression in tumor cells. **A,** Representative IHC images of KP and KPP tumors stained with MHC-II. Scale bar, 100 μm. **B,** Flow cytometry detection of MHC-II in KP and KPP cells. **C,** Quantification of B (n = 3). The averages of MHC-II in the histogram of the gated populations are normalized to KP and shown as Relative Level (mean ± SD). Also see S1 Appendix. **D,** ELISA detection of IFN-γ in KP and KPP cells (n = 6). Data are normalized to KP and shown as mean ± SD. **E,** Flow cytometry detection of MHC-II in KP cells treated with PLK1i (50 nM BI, 50 nM GSK, 200 nM ONV) for 48 hours. **F,** Quantification of E (n = 3). The averages of MHC-II in the histogram of the gated populations are normalized to Ctrl and shown as Relative Level (mean ± SD). Also see S1 Appendix. **G,** Flow cytometry detection of MHC-II in KPP cells treated with PLK1i (50 nM BI, 50 nM GSK, 200 nM ONV) for 48 hours. **H,** Quantification of G (n = 3). The averages of MHC-II in the histogram of the gated populations are normalized to Ctrl and shown as Relative Level (mean ± SD). Also see S1 Appendix. For these results, an unpaired one-sided t test was used. **I,** Flow cytometry detection of MHC-II in H358 cells 48 hours after transfection with siRNA control or siRNA targeting PLK1. **J,** Quantification of I (n = 3). The averages of MHC-II in the histogram of the gated populations are normalized to Ctrl and shown as Relative Level (mean ± SD). Also see S1 Appendix. Data are normalized to Ctrl and shown as mean ± SD. **K,** Flow cytometry detection of MHC-II in H358 cells with dose escalation of PLK1 inhibitor ONV for 48 hours. **L,** Quantification of K (n = 3). The averages of MHC-II in the histogram of the gated populations are normalized to untreated group (0) and shown as Relative Level (mean ± SD). Also see S1 Appendix. *, p < 0.05. **, p < 0.01. ***, p < 0.001.

It has been reported that MHC-II can also express on the surface of cancer cells, and this phenotype is associated with improved response to immunotherapy and better survival of cancer patients [46–49]. However, this phenotype was unexplored in lung cancer. Based on these established facts and our observations that PLK1 was negatively associated with MHC-II, we aimed to explore whether PLK1 affected the expression of MHC-II in LUAD tumors. We first detected MHC-II expression by IHC staining on tumor slides prepared from KP and KPP mice, and the results indeed showed that both tumor cells expressed MHC-II, with more striking expression in KP tumors (**Fig 5A**). This was consistent with the results observed in immune cells that high PLK1 led to lower MHC-II level. Next, we performed flow cytometry analysis of MHC-II level in KP and KPP cell lines, and the results verified the lower expression of MHC-II in KPP cells (**Fig 5B and 5C**). Since MHC-II expression in cancer cells depends on the interferon gamma (IFN-γ) [50], we performed ELISA experiment to detect intracellular IFN-γ levels between KP and KPP cells. As expected, KPP cells had a lower level of IFN-γ compared to KP (**Fig 5D**), consistent with its inferior expression of MHC-II. To direct assess the effect of PLK1, we treated KP and KPP with three PLK1 inhibitors (PLK1i), including BI-2536 (BI), GSK461364A (GSK) and Onvansertib (ONV). In both cells, treatment with PLK1i consistently elevated the level of MHC-II (**Fig 5E–5H**), confirming the negative regulation of MHC-II by PLK1. To assess the human side, we utilized human H358 LUAD cell line to see whether PLK1 played a similar role. We first transfected H358 cells with siRNA targeting PLK1, and the depletion of PLK1 elevated MHC-II as expected (**Fig 5I and 5J**). Dose escalation of one PLK1 inhibitor ONV also showed a gradual increase in MHC-II expression (**Fig 5K and 5L**), reassuring the effect of PLK1. Cumulatively, these data supported the conclusion that PLK1 also regulated MHC-II in LUAD cancer cells.

### Clinical assessment of PLK1’s regulation of MHC-II and M2 polarization

**Fig 6:**
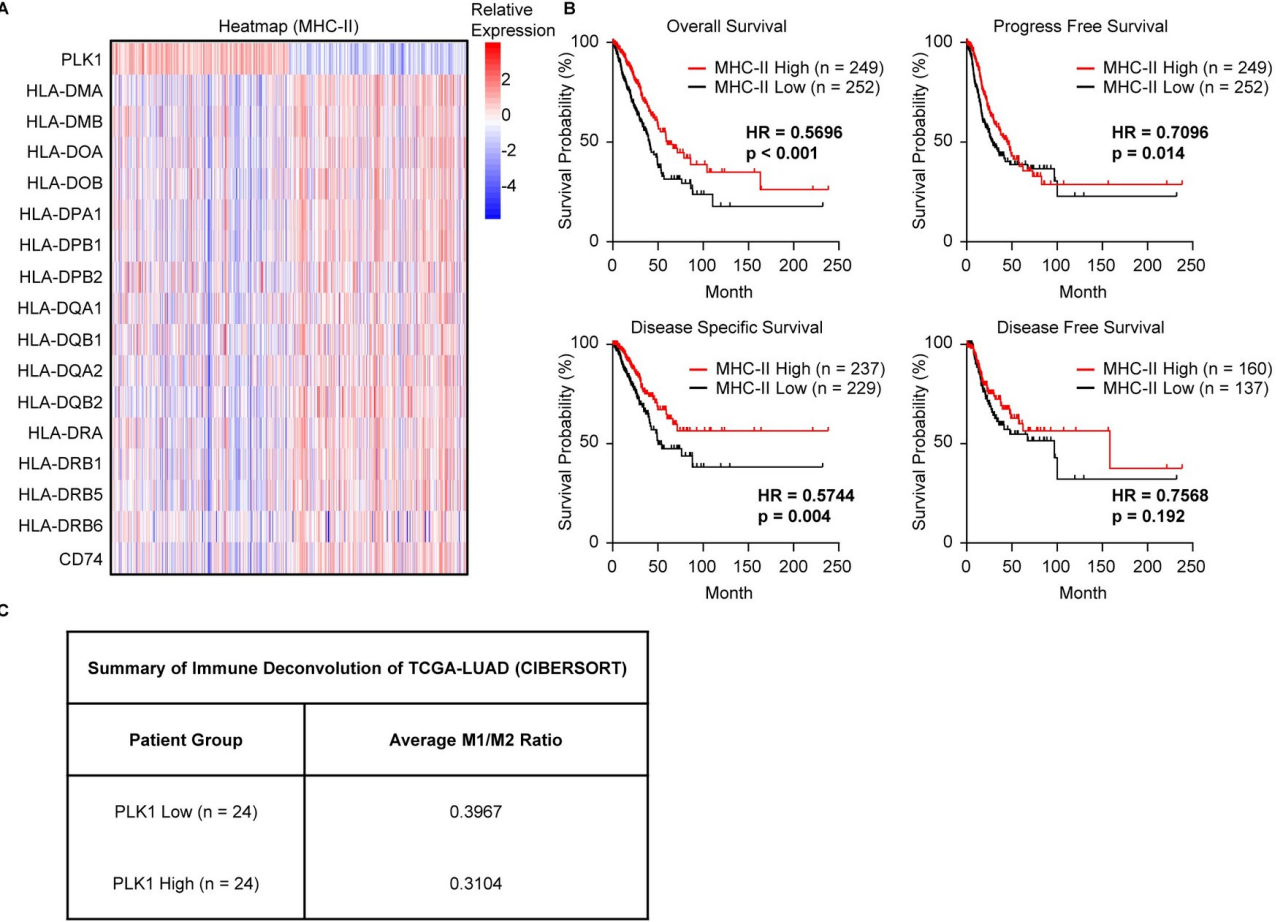
Clinical evidence of PLK1 in suppressing MHC-II in lung cancer. **A,** Heatmap of MHC-II signature genes in TCGA-LUAD patients separated into high PLK1 and low PLK1 groups. Patients are clustered based on the median expression (zscores of Log_2_RSEM+1) of PLK1. **B,** Survival curves of TCGA-LUAD patients separated into MHC-II High and MHC-II Low groups. Log_2_RSEM+1 of MHC-II signature genes in A are averaged to get signature scores. Patients are clustered based on the median value of signature scores. For these results, a Log-rank test was used. **C,** Immune deconvolution of bulk RNA-seq data of TCGA-LUAD patients with TP53 mutations and KRAS mutations by CIBERSORT algorithm. The median expression of PLK1 (RSEM) is used to separate patients into PLK1 Low and PLK1 High groups. For each patient, the M1/M2 ratio is calculated, and the results are shown as the average M1/M2 ratio of all patients in that group.

We’ve previously demonstrated that high PLK1 was associated with worse patients’ outcomes [19], and this was consistent with the data that PLK1 suppressed MHC-II and promoted M2 polarization presented in this study. To evaluate the translational values of our findings, we analyzed the TCGA-LUAD dataset. We found that high PLK1 was associated with low levels of MHC-II member genes (**Figs 6A and S6**). Using this MHC-II gene signature, we calculated the MHC-II signature scores of all patients and used these signature scores to re-classify patients into high MHC-II and low MHC-II groups, then performed survival analysis of those patients. We found that patients with high MHC-II scores had better survival outcomes compared to patients with low MHC-II scores (**Fig 6B**). We also performed the immune deconvolution of bulk TCGA-LUAD RNA-seq using patients with TP53 mutations and KRAS mutations by CIBERSORT algorithm [33]. We found that patients with high PLK1 displayed lower M1/M2 ratio, indicating that these patients had more M2 macrophages in the bulk tumor samples (**Fig 6C and S7 Table**). All these results were consistent with our previous findings and supported the notion that the negative regulation of MHC-II and the M2 polarization by PLK1 might promote LUAD progression and result in poor outcomes.

## Discussion

**Fig 7:**
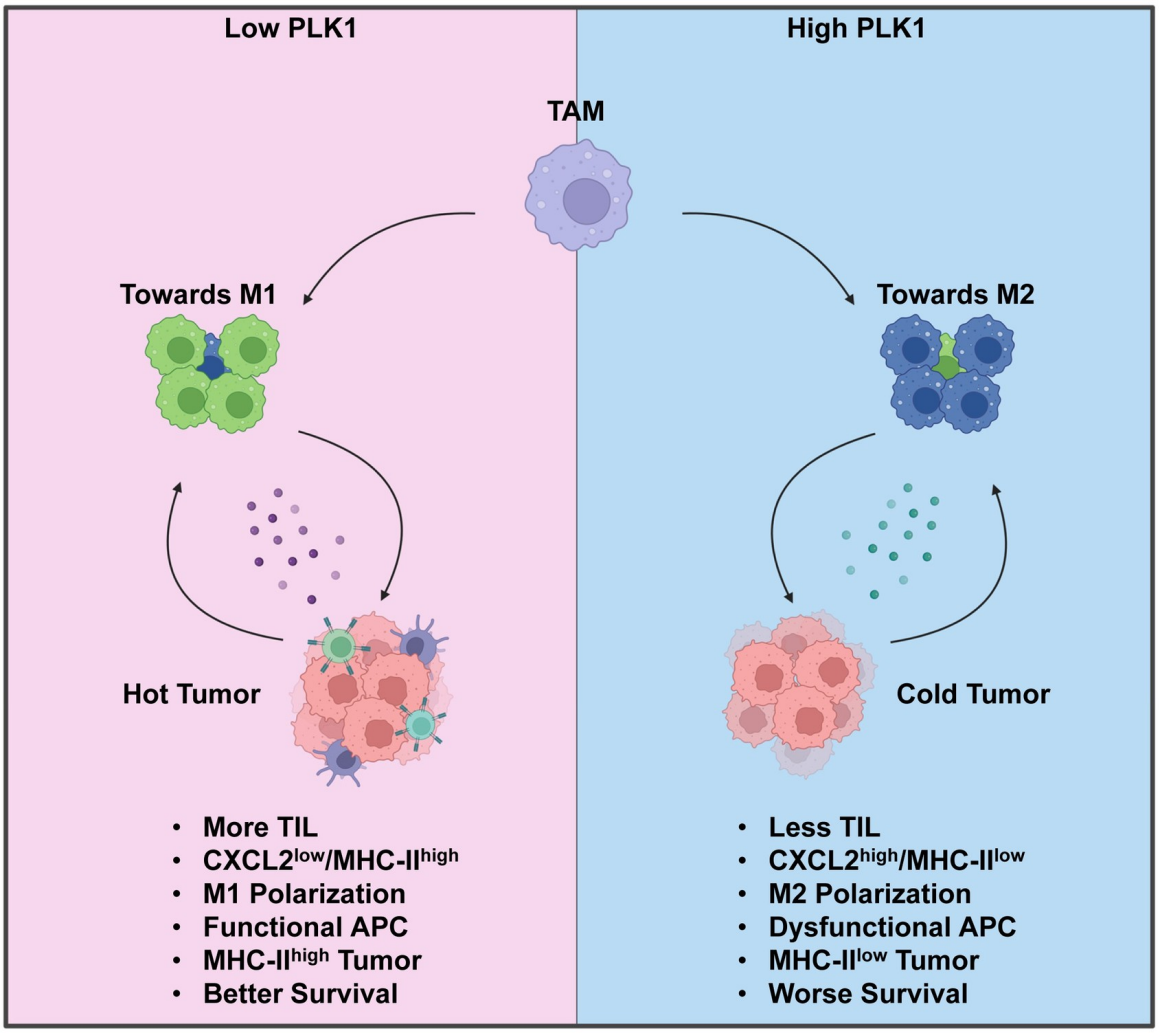
Summary of working model. Under low PLK1 condition, there is low level of CXCL2 secreted from tumors. Tumor microenvironment is “hot”, characterized by increased tumor infiltrating lymphocytes, M1 polarization of TAM and functional APC. Cancer cells are MHC-II high and this feature is associated with better survival of patients. Under high PLK1 condition, there is increased secretion of CXCL2 from tumors. Tumor microenvironment is “cold”, characterized by low levels of tumor infiltrating lymphocytes, M2 polarization of TAM and dysfunctional APC. Cancer cells are MHC-II low and patients have worse survival outcomes. Created with BioRender.com.

Here in this study, we render pioneering data to support the fact that PLK1 promotes an immunosuppressive TME in LUAD (**Fig 7**). Under low PLK1 condition, TME is characterized by a higher proportion of tumor infiltrating lymphocytes and a lower level of CXCL2, and this condition is associated with more M1 polarization and functional antigen presentation pathway. In addition, tumors express more MHC-II and this feature is associated with better patient’s survival. Under high PLK1 condition, TME is characterized by low tumor infiltrating lymphocytes and high secreted CXCL2, which promotes M2 polarization and disrupts antigen processing and presentation. Tumors in this situation also express a lower level of MHC-II, which is associated with worse outcomes of patients. The differences in TME shown in high PLK1 and low PLK1 conditions suggest that PLK1 is an important modulator of TME, and targeting PLK1 may be a practical therapeutic intervention in LUAD treatment.

Although the emergence of immunotherapy has expanded the treatment options for lung cancer patients, the efficacy is questionable due to resistance to immunotherapy. Thus, identifying mechanisms behind this harmful condition is urgently needed. Here, we report a novel mechanism of PLK1 in cancer promotion. Previously, PLK1 has been largely considered as a canonical cell cycle regulator in the past decades. However, recent studies have gradually uncovered the tumor-promoting functions of this kinase. The fact that PLK1 suppresses anti-tumor in LUAD is intriguing given that immunotherapy is one of the major therapeutic approaches for lung cancer. The major reasons of resistance to immunotherapies are the scarcity of tumor-infiltrating immune cells and existence of immune checkpoints [51]. We show that high PLK1 is associated with lower infiltrating T cells and NK cells, and this feature may be harmful to anti-tumor response in LUAD. The decreased infiltration of T cells and NK cells may be due to the increased M2 polarization from TAM, which is a well-established suppressor of their functions. Besides, functional antigen presentation in TME is the key to the success of anti-tumor immune response, and immune evasion through suppressing this critical process significantly reduces the efficacy of immunotherapy [36], which is also seen in our model. The novelty of our study lies in the fact we first demonstrate that PLK1 promotes M2 polarization and inhibits antigen presentation pathway by down-regulating MHC-II in professional antigen-presenting cells, which is the key molecule responsible for processing and presentation of tumor-specific neoantigens. Besides, the regulation of MHC-II can be extended to cancer cells, where high PLK1 is also associated with low MHC-II expression. The expression of MHC-II in tumors is a good biomarker for better survival of patients [46–49]. However, the function of MHC-II in cancer cells is unclear. Given that MHC-II is mainly responsible for antigen presentation, the fact that MHC-II expressing in cancer cells may be involved in this process by functionally mimicking antigen presentation process and priming CD4 T cells, which directly bind to MHC-II to activate immune response, and this idea has been supported by the discovery of certain MHC-II specific neoantigens in tumor cells and their necessities in promoting successful anti-tumor response [49]. Considering the negative regulation of MHC-II by PLK1, our study further supports this notion in LUAD. Despite that, the mechanistic part behind this regulation is missing and requires further exploration.

Given the predominant immunosuppressive functions of PLK1 in LUAD and other cancer types, treatment focuses on targeting PLK1 may decelerate tumor growth by boosting immune response in TME. However, it is noteworthy that PLK1 may be negatively correlated with PD-L1 in certain situations [20,21], devaluing the monotherapy with PLK1 inhibitor as this approach will unexpectedly elevate immune checkpoints. Thus, combination treatment with PLK1i and immune checkpoint blockade should exhibit premium effect, and this assumption remains to be investigated by future studies.

## Supporting information

S1 Fig scRNA-seq analysis. **A,** Feature plots of marker genes used for classification of immune cells (*Ptprc*) and non-immune cells (*Krt18*). Color scale represents the relative expression levels of genes. **B,** UMAP of immune and non-immune cells in KP and KPP. **C,** Comparison of immune and non-immune cell proportions between KP and KPP (n = 3). Data are shown as mean ± SD. **D,** UMAP of immune cell clusters in KP and KPP. **E,** Heatmap of top 5 signature genes of each cluster in D. Also see S1 Table for full gene list. **F,** Proportions of each cluster of immune cells in KP and KPP. **G,** Comparison of immune cell cluster proportions between KP and KPP. Statistical method: two-sample binomial test. ***, FDR < 0.001.
(PDF)

S2 Fig scRNA-seq analysis of T cell and NK cell populations. **A,** Feature plots of marker genes used for roughly identifying different T cell and NK cell populations. Color scale represents the relative expression levels of genes. **B,** UMAP projection of T cell and NK cell populations. **C,** Comparison of proportions (in total immune cells) of T cell and NK cell populations denoted in B between KP and KPP (n = 3). Data are shown as mean ± SD. *, p < 0.05. **, p < 0.01. ***, p < 0.001.
(PDF)

S3 Fig GSEA results of immune cell populations. Significant pathways (KPP vs KP, FDR < 0.1) in the indicated immune cell populations are shown.
(PDF)

S4 Fig RNA-seq analysis of KP and KPP cells. **A,** STRING analysis of pathway network associated with CXCL2 in human and mice. **B,** GSEA table of two potential pathways regulating CXCL2 secretion in KPP cells, identified by our previously published RNA-seq data (GSE206644, KPP vs KP). FDR < 0.05 indicates a significance.
(PDF)

S5 Fig Coculture experiments of dendritic cells. **A,** Flow cytometry analysis of indicated genes in dendritic cells cocultured with KP or KPP at the indicated ratio for 48 hours. **B,** Quantification of A (n = 3). The percentage of positive cells after gating is shown as mean ± SD. Also see S1 Appendix. **C,** Flow cytometry analysis of dendritic cells cocultured with conditioned medium from KP or KPP for 48 hours, with or without recombinant *Cxcl2* (2 ug/ml) or CXCR1/CXCR2 inhibitor SX-682 (1 μM) treatment. **D,** Quantification of C (n = 3). The percentage of positive cells after gating is shown as mean ± SD. Also see S1 Appendix. **E,** Flow cytometry analysis of dendritic cells cocultured with conditioned medium from KP or KPP for 48 hours, with or without anti-*Cxcl2* neutralization antibodies (5 ug/ml) treatment. **F,** Quantification of E (n = 3). The percentage of positive cells after gating is shown as mean ± SD. Also see S1 Appendix. *, p < 0.05. **, p < 0.01. ***, p < 0.001.
(PDF)

S6 Fig Correlation analysis of PLK1 and MHC-II genes. Patients’ data (Log_2_RSEM) are collected from TCGA-LUAD. r_s_, spearman correlation coefficient.
(PDF)

S1 Table Differentially expressed genes for all immune cell clusters. (XLSX)

S2 Table Differentially expressed genes for identified immune cell populations. (XLSX)

S3 Table Differentially expressed genes for NK/T cell populations. (XLSX)

S4 Table Differentially expressed genes for macrophages. (XLSX)

S5 Table Gene signatures for M1 and M2 macrophages. (XLSX)

S6 Table Gene Set Enrichment Analysis of identified immune cell populations. (XLSX)

S7 Table Immune deconvolution of TCGA-LUAD bulk RNA-seq by CIBERSORT. (XLSX)

S8 Table Reagents information. (XLSX)

S9 Table Numeric values for rebuilding figures. (XLSX)

S1 Appendix Gating strategy of flow cytometry experiments. (PDF)
