# Supplementary figures and images for: Single-cell analysis identifies PLK1 as a driver of immunosuppressive tumor microenvironment in LUAD
Source: PLoS Genet. 2024 Jun 17;20(6):e1011309. doi: 10.1371/journal.pgen.1011309 (PMC11182521; doi:10.1371/journal.pgen.1011309)

## Figure S1

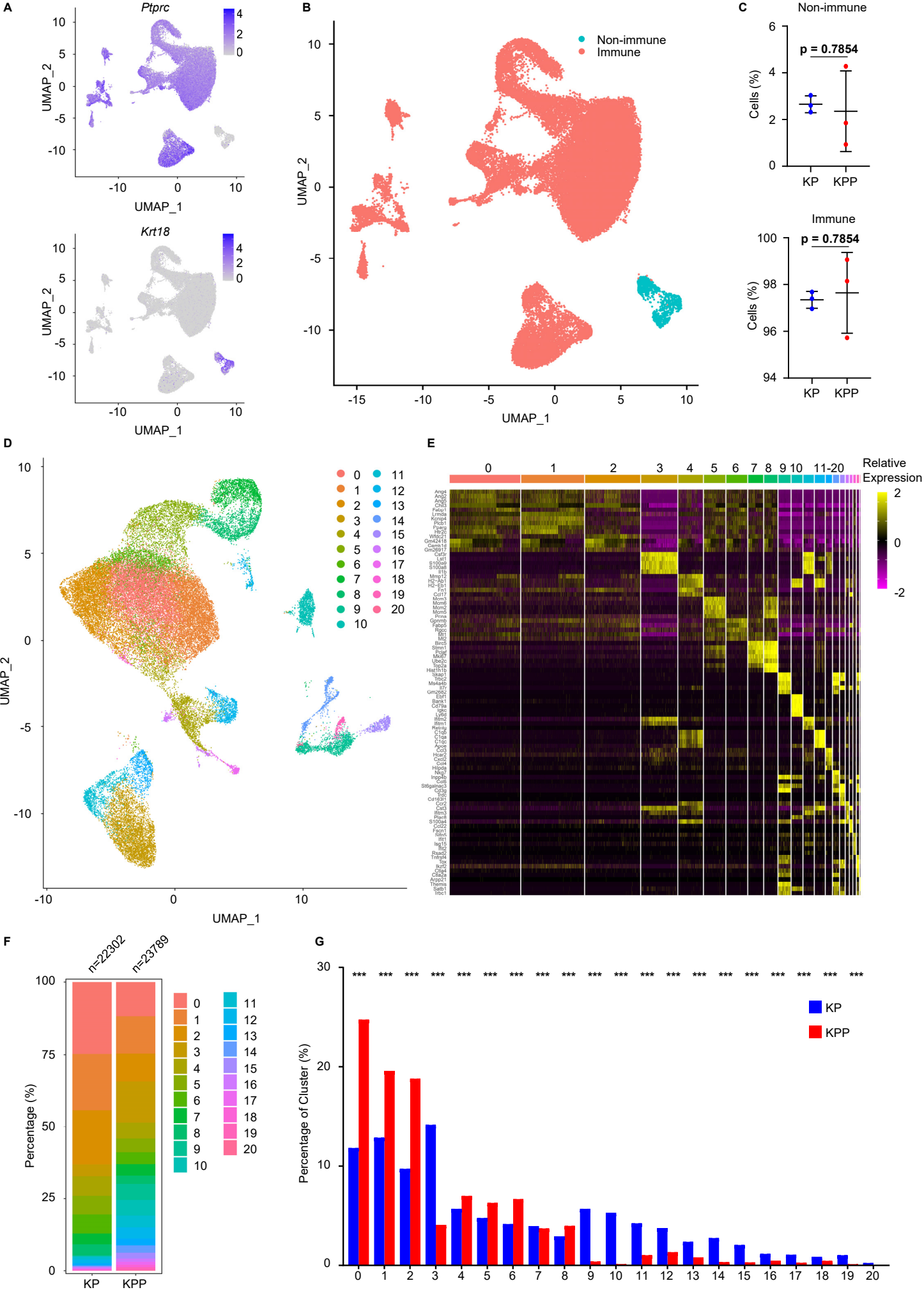

Supplement: S1 Fig — A, Feature plots of marker genes used for classification of immune cells (Ptprc) and non-immune cells (Krt18). Color scale represents the relative expression levels of genes. B, UMAP of immune and non-immune cells in KP and KPP. C, Comparison of immune and non-immune cell proportions between KP and KPP (n = 3). Data are shown as mean ± SD. D, UMAP of immune cell clusters in KP and KPP. E, Heatmap of top 5 signature genes of each cluster in D. Also see S1 Table for full gene list. F, Proportions of each cluster of immune cells in KP and KPP. G, Comparison of immune cell cluster proportions between KP and KPP. Statistical method: two-sample binomial test. ***, FDR < 0.001. (PDF) [file pgen.1011309.s001.pdf]

**Figure S2**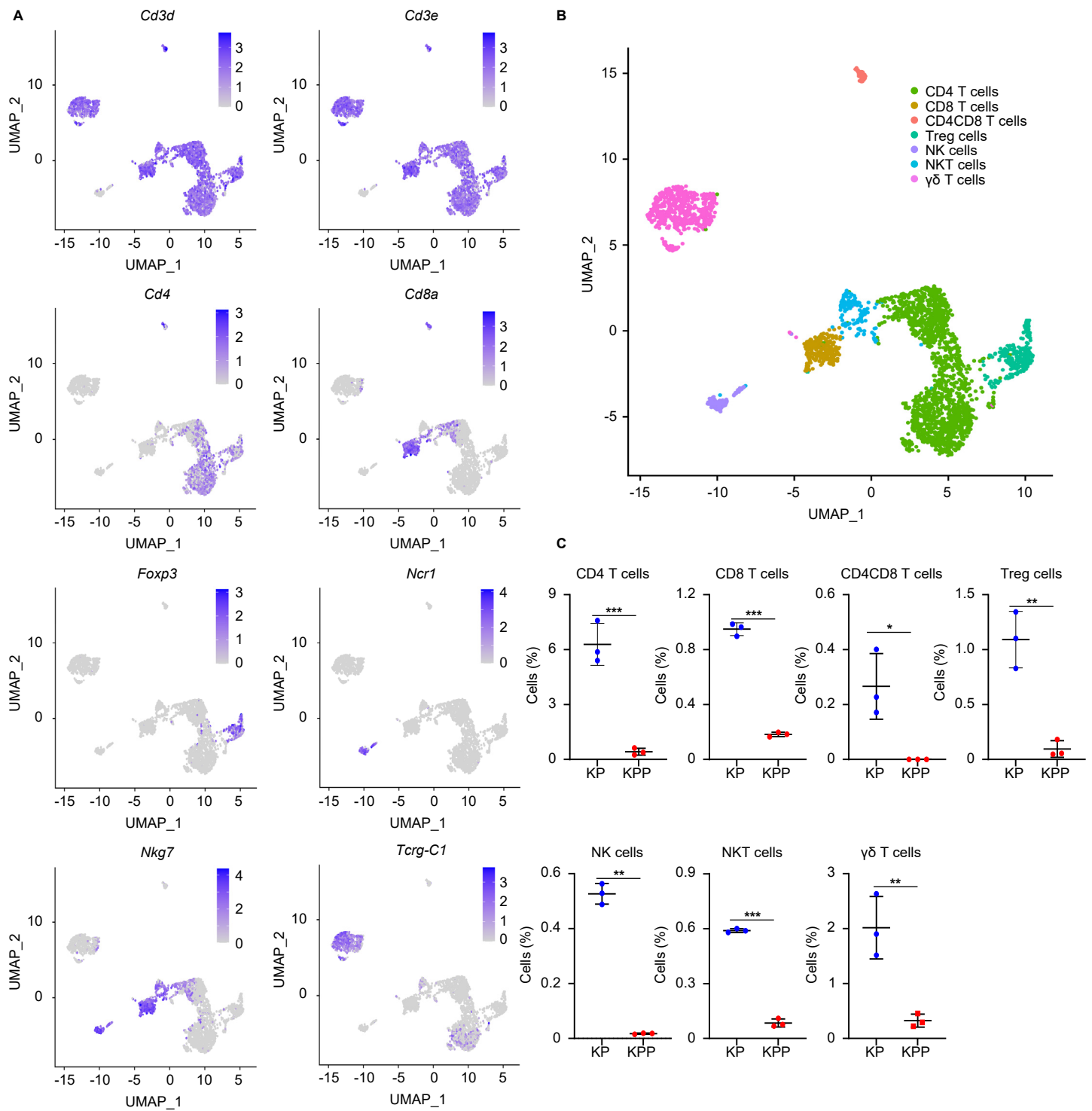

Supplement: S2 Fig — A, Feature plots of marker genes used for roughly identifying different T cell and NK cell populations. Color scale represents the relative expression levels of genes. B, UMAP projection of T cell and NK cell populations. C, Comparison of proportions (in total immune cells) of T cell and NK cell populations denoted in B between KP and KPP (n = 3). Data are shown as mean ± SD. *, p < 0.05. **, p < 0.01. ***, p < 0.001. (PDF) [file pgen.1011309.s002.pdf]

**Figure S3**

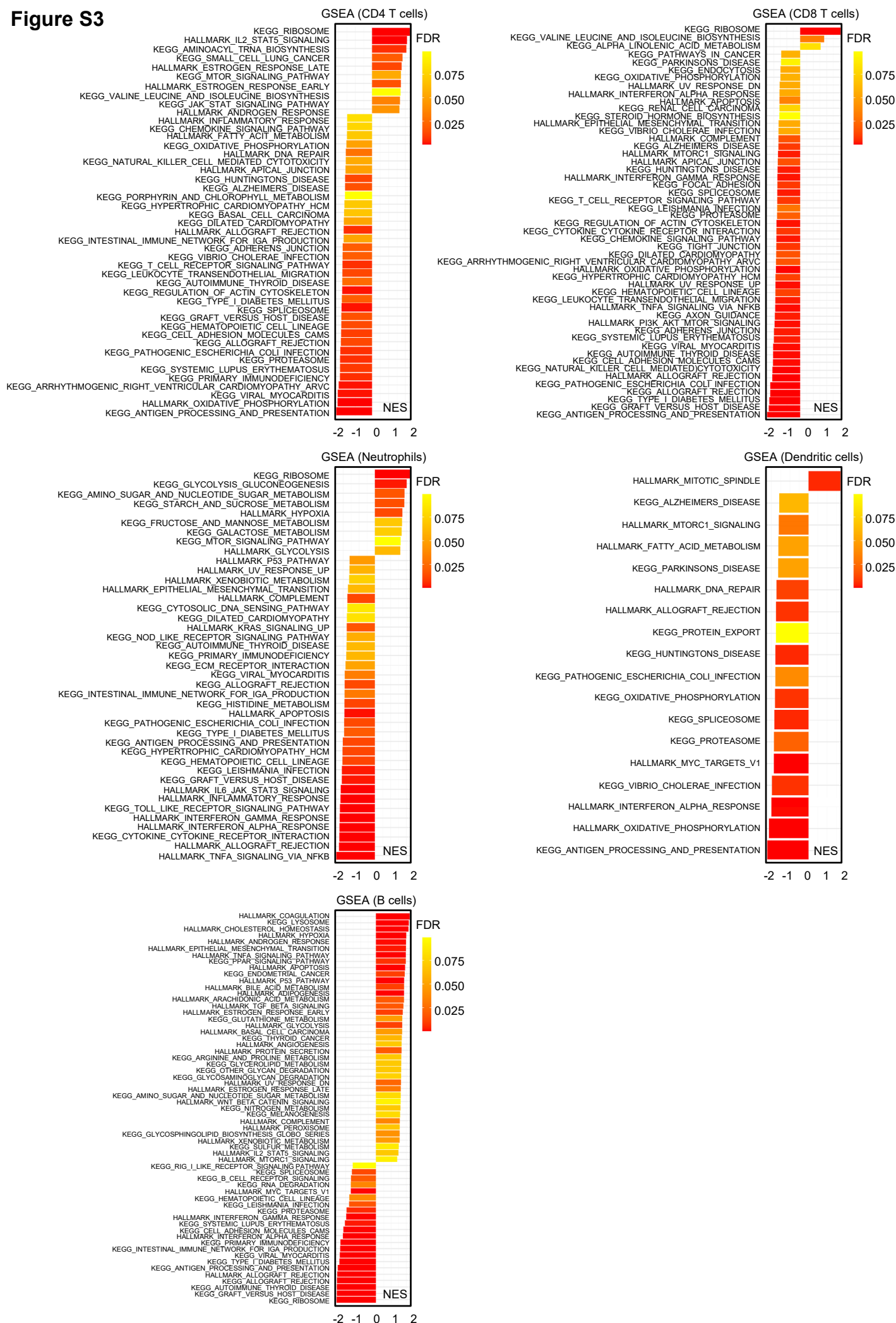

Supplement: S3 Fig — Significant pathways (KPP vs KP, FDR < 0.1) in the indicated immune cell populations are shown. (PDF) [file pgen.1011309.s003.pdf]

**Figure S5**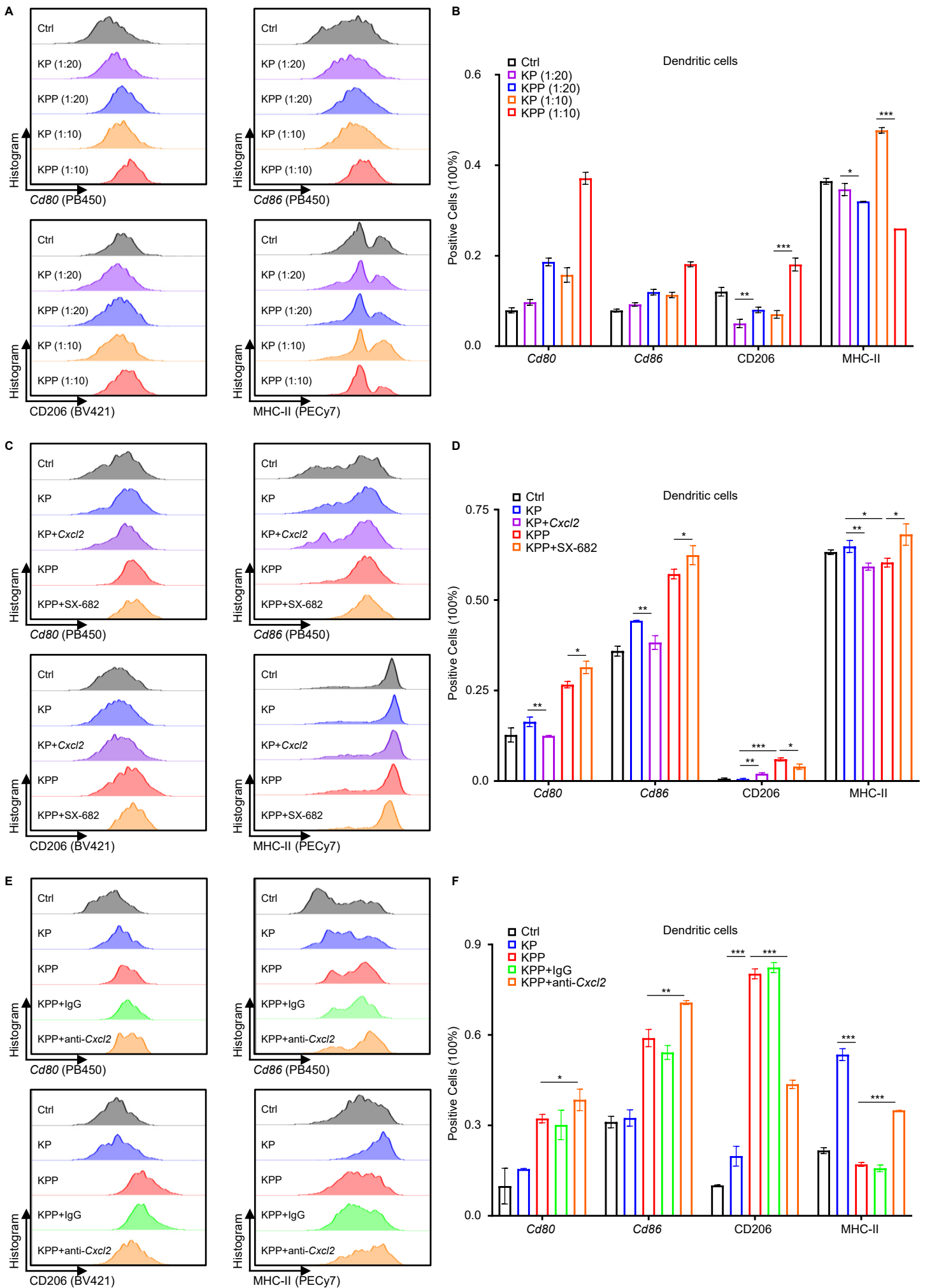

Supplement: S5 Fig — A, Flow cytometry analysis of indicated genes in dendritic cells cocultured with KP or KPP at the indicated ratio for 48 hours. B, Quantification of A (n = 3). The percentage of positive cells after gating is shown as mean ± SD. Also see S1 Appendix. C, Flow cytometry analysis of dendritic cells cocultured with conditioned medium from KP or KPP for 48 hours, with or without recombinant Cxcl2 (2 ug/ml) or CXCR1/CXCR2 inhibitor SX-682 (1 μM) treatment. D, Quantification of C (n = 3). The percentage of positive cells after gating is shown as mean ± SD. Also see S1 Appendix. E, Flow cytometry analysis of dendritic cells cocultured with conditioned medium from KP or KPP for 48 hours, with or without anti-Cxcl2 neutralization antibodies (5 ug/ml) treatment. F, Quantification of E (n = 3). The percentage of positive cells after gating is shown as mean ± SD. Also see S1 Appendix. *, p < 0.05. **, p < 0.01. ***, p < 0.001. (PDF) [file pgen.1011309.s005.pdf]

**Figure S6**

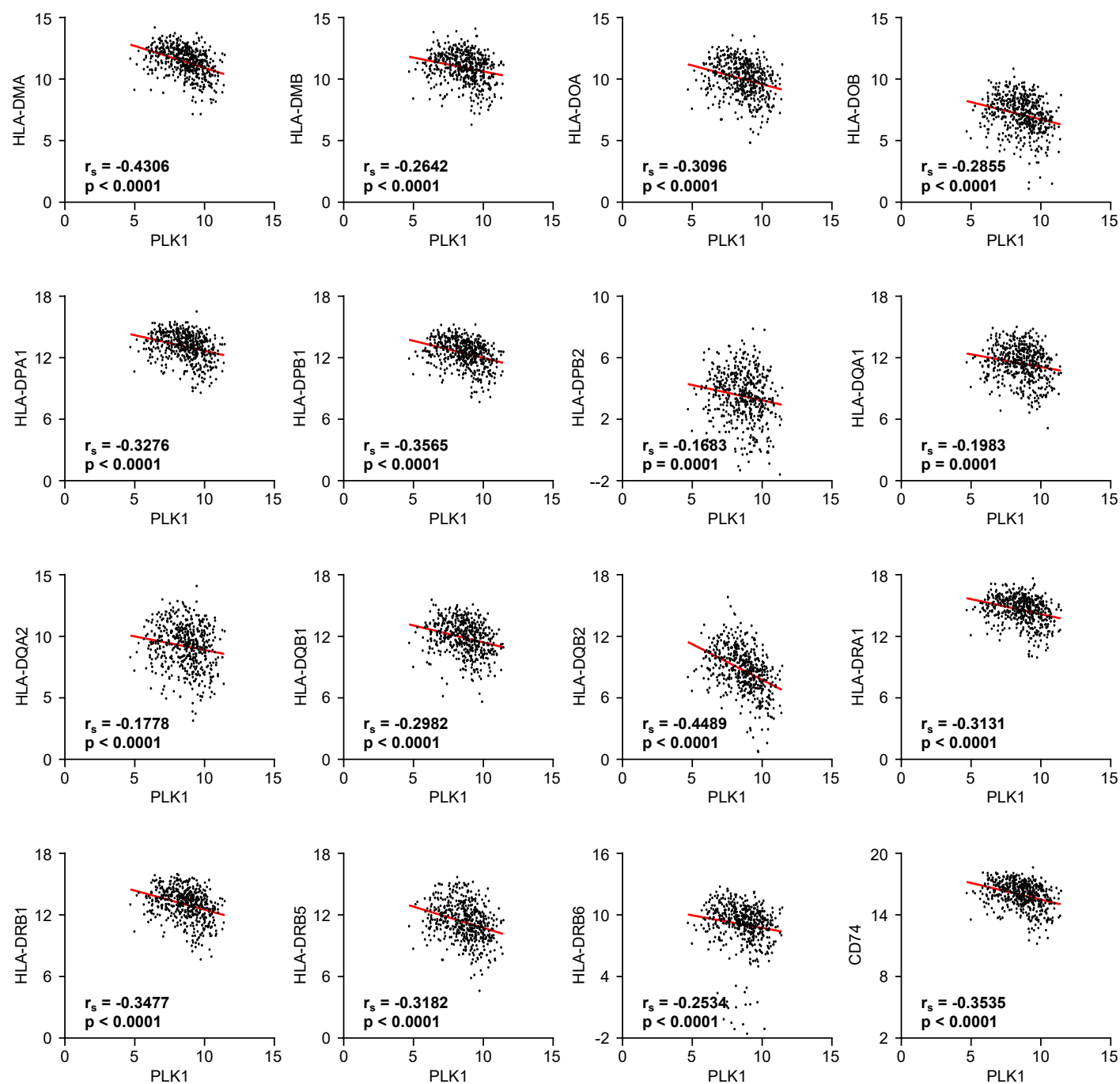

Supplement: S6 Fig — Patients’ data (Log2RSEM) are collected from TCGA-LUAD. rs, spearman correlation coefficient. (PDF) [file pgen.1011309.s006.pdf]

3C

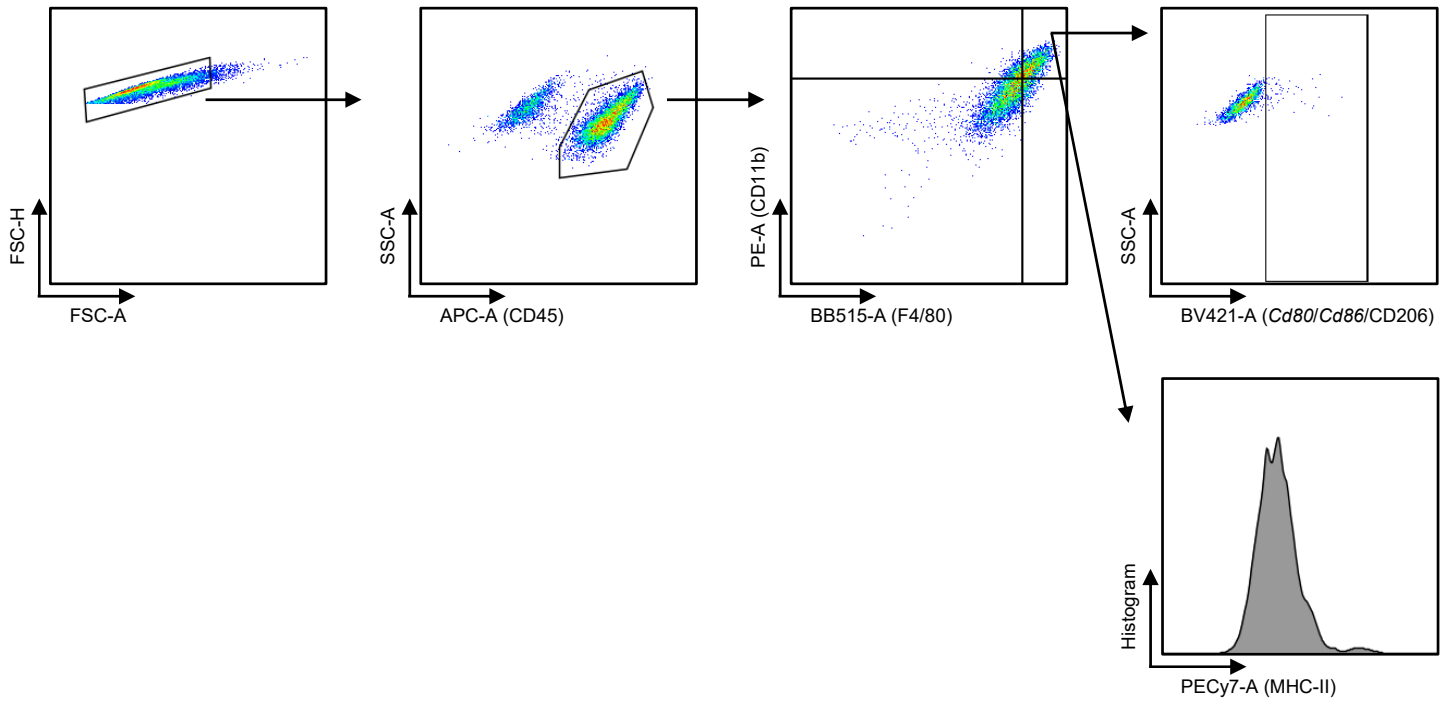

4C, 4E

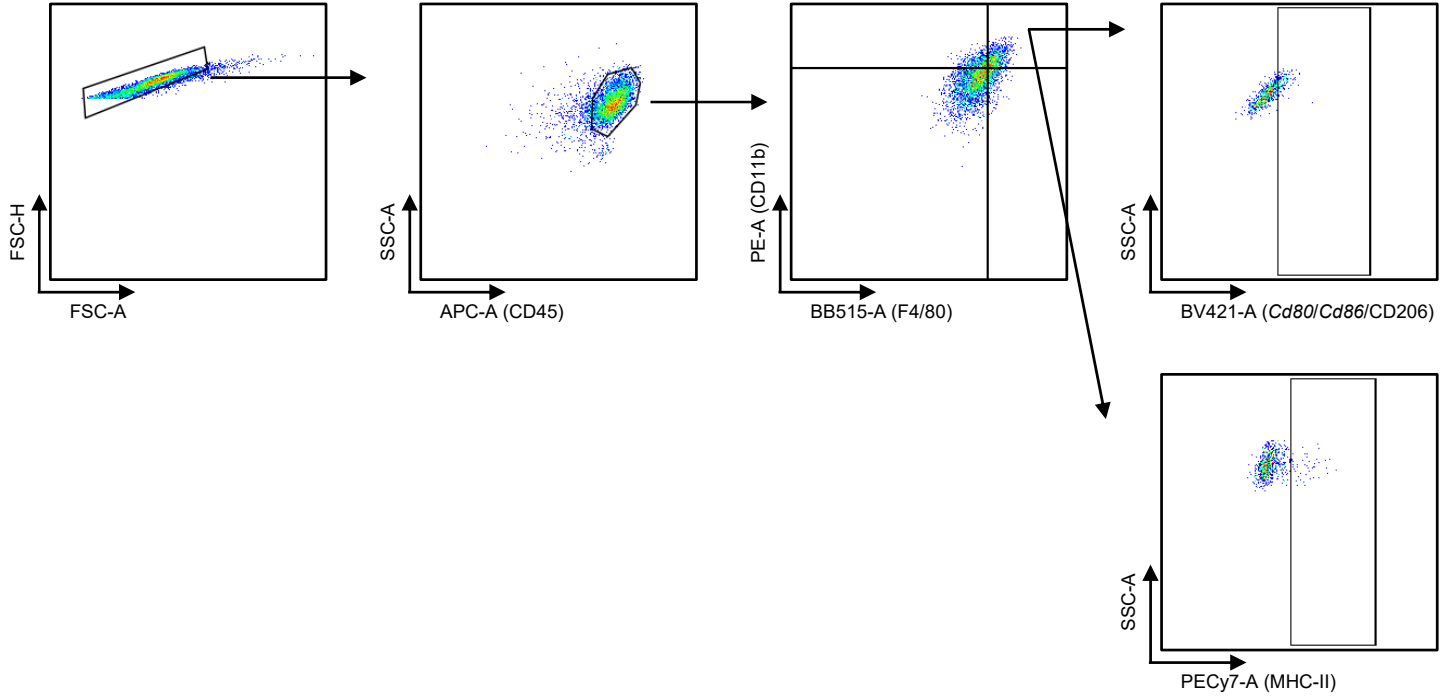

5B, 5E, 5G

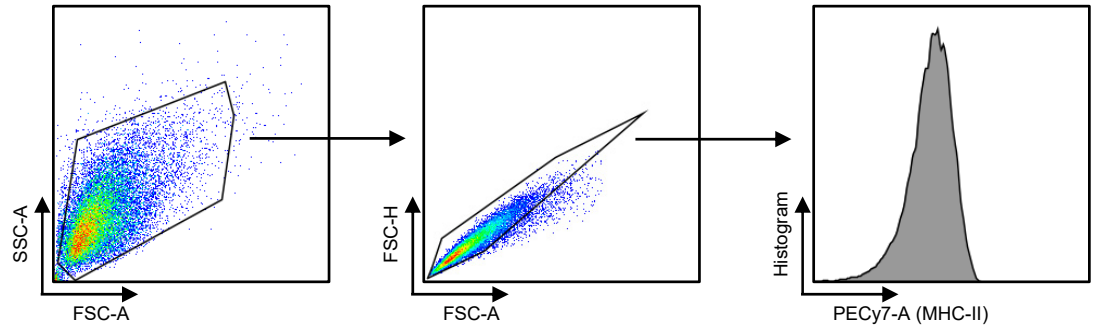

5I, 5K

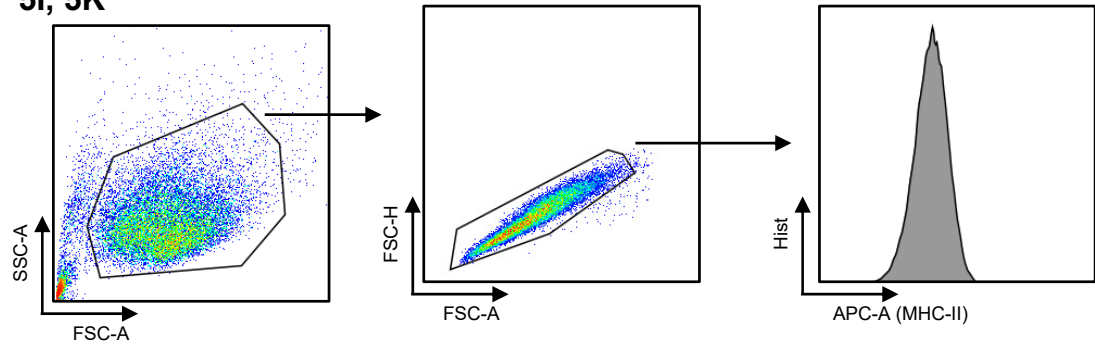

**S5A**

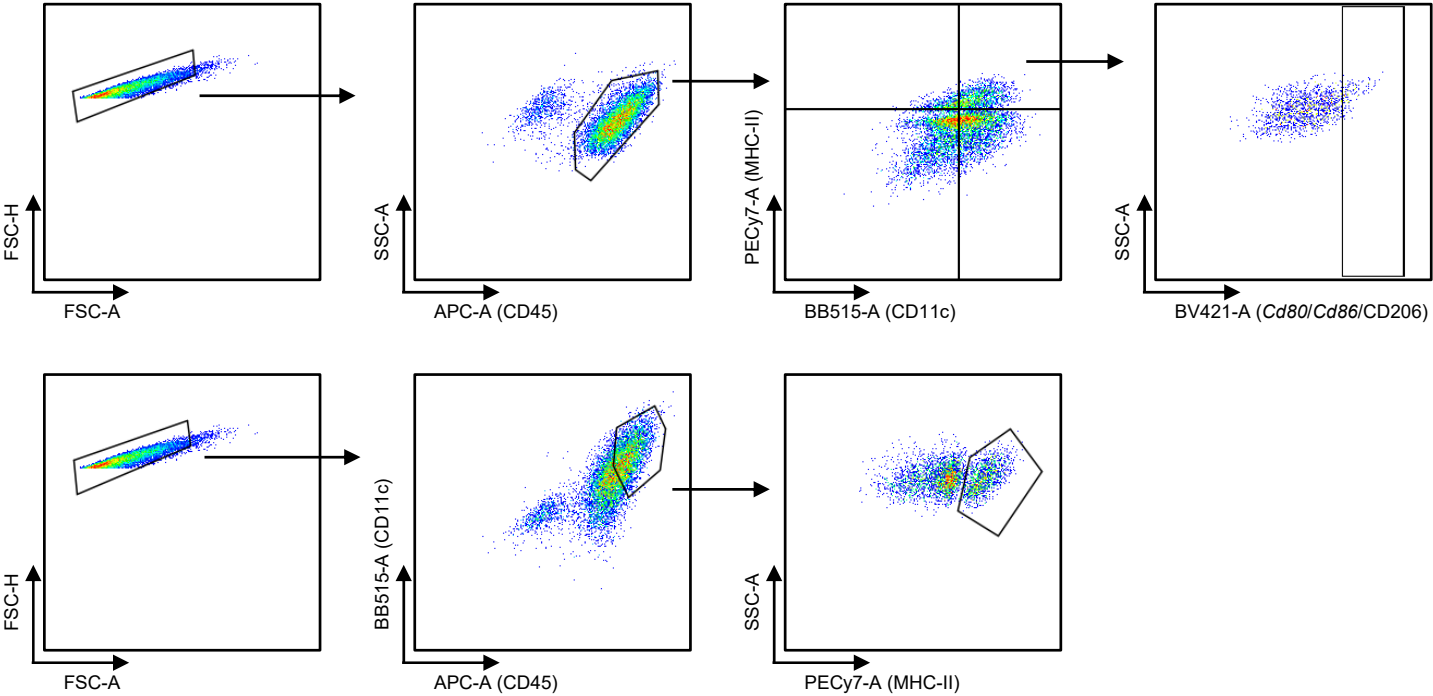

**S5C, S5E**

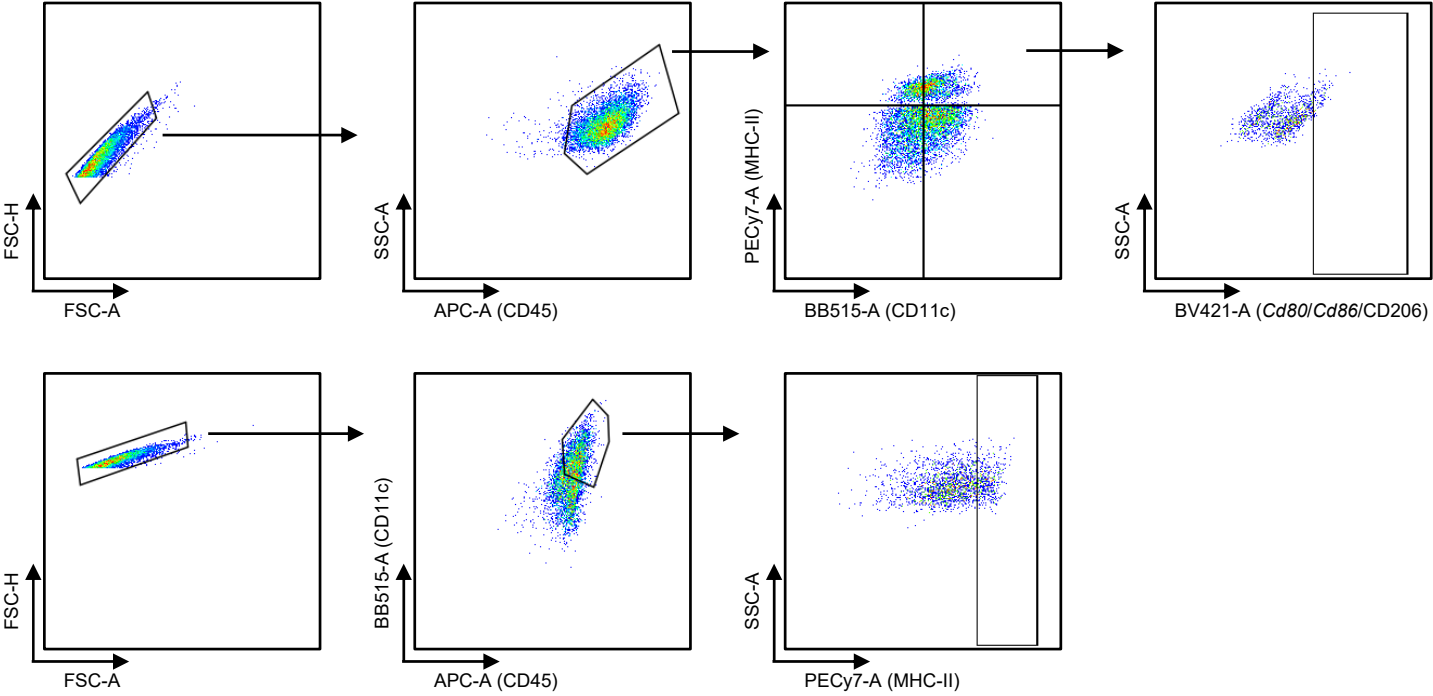

Supplement: S1 Appendix — (PDF) [file pgen.1011309.s016.pdf]
