# Supplementary material for: Single-cell analysis identifies PLK1 as a driver of immunosuppressive tumor microenvironment in LUAD
Source: PLoS Genet. 2024 Jun 17;20(6):e1011309. doi: 10.1371/journal.pgen.1011309 (PMC11182521; doi:10.1371/journal.pgen.1011309)
Supplement: S4 Fig — A, STRING analysis of pathway network associated with CXCL2 in human and mice. B, GSEA table of two potential pathways regulating CXCL2 secretion in KPP cells, identified by our previously published RNA-seq data (GSE206644, KPP vs KP). FDR < 0.05 indicates a significance. (PDF) [file pgen.1011309.s004.pdf]

Figure S4

A

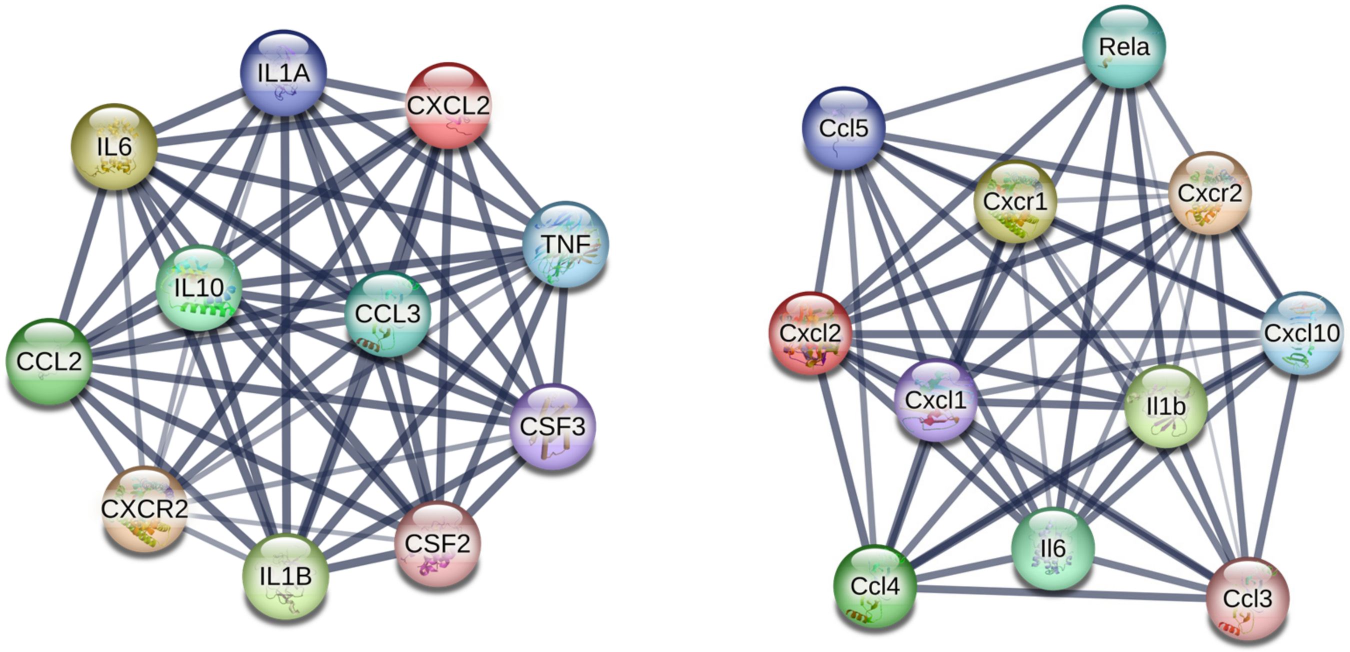

B

GSEA of RNA-seq: GSE206644 (KPP vs KP)		
	**HALLMARK_ IL6_JAK_STAT3_SIGNALING**	**HALLMARK_ TNFA_SIGNALING_VIA_NFKB**
**NES**	1.56	1.52
**FDR**	**0.023**	**0.028**
